# Nationwide implementation of heart failure therapies: National Heart Failure Center Accreditation Program (HF‐CAP) in China

**DOI:** 10.1002/ejhf.70035

**Published:** 2025-09-22

**Authors:** Jingmin Zhou, Xuejuan Jin, Yamei Xu, Zhonglei Xie, Xiaotong Cui, Yanyan Wang, Hua Wang, Xinli Li, Yugang Dong, Yuhua Liao, Weimin Li, Alexandre Mebazaa, Jiefu Yang, Junbo Ge, on behalf of the Heart Failure Center Accreditation Program Investigators

**Affiliations:** ^1^ Department of Cardiology Zhongshan Hospital, Fudan University, Shanghai Institute of Cardiovascular Diseases Shanghai China; ^2^ Department of Epidemiology Zhongshan Hospital, Fudan University, Shanghai Institute of Cardiovascular Diseases Shanghai China; ^3^ Department of Cardiology Beijing Hospital, National Center of Gerontology, Institute of Geriatric Medicine, Chinese Academy of Medical Sciences Beijing China; ^4^ Department of Cardiology The First Affiliated Hospital of Nanjing Medical University Jiangsu China; ^5^ Department of Cardiology, Center for Translational Medicine Institute of Precision Medicine, The First Affiliated Hospital, Sun Yat‐sen University Guangzhou China; ^6^ NHC Key Laboratory of Assisted Circulation (Sun Yat‐sen University) Guangzhou China; ^7^ Department of Cardiology Union Hospital Affiliated to Tongji Medical College of Huazhong University of Science and Technology Hubei China; ^8^ Department of Cardiology First Affiliated Hospital of Harbin Medical University Heilongjiang China; ^9^ Université Paris Cité, Hôpitaux Universitaires Saint Louis Lariboisière, APHP Paris France

**Keywords:** Guideline‐directed medical therapy, Heart failure, Hospitalization, National Heart Failure Center Accreditation Program, Readmission

## Abstract

**Aims:**

Implementing optimal guideline‐directed medical therapy is still challenging in patients with heart failure (HF). This prospective study assessed the benefits of large‐scale, nationwide, multi‐annual implementation of HF therapies in China.

**Methods and results:**

This longitudinal, pre‐post comparison design included patients in hospitals accredited by the National Heart Failure Center Accreditation Program (HF‐CAP). Patients were divided into four groups: 6–12 months before accreditation (Pre); >0 –≤12 months after accreditation (Y1); >12–≤24 months after accreditation (Y2), and >24 months after accreditation (Y2+). The primary endpoint was 1‐year composite HF readmission and/or cardiovascular death. Secondary endpoints included 1‐year HF readmission alone, 1‐year cardiovascular death alone, and association between phone calls and/or visits and outcomes. Overall, 408 073 patients with HF from 646 centres were included. After HF‐CAP accreditation, more patients with HF were treated following discharge. Compared with the Pre group, risk of meeting the primary endpoint decreased in Y1 and was incrementally lower in Y2 and Y2+: fully adjusted odds ratios (OR) and 95% confidence intervals (CIs) were 0.893 (0.871–0.916), 0.855 (0.830–0.880) and 0.720 (0.695–0.745), respectively (all *p* < 0.0001). Risk of HF readmission alone reduced from Y1 onwards (OR 0.865 [95% CI 0.841–0.891]). Risk of cardiovascular death reduced from Y2 onwards (OR 0.942 [95% CI 0.904–0.983]). Phone calls had little association with patient outcomes; however, face‐to‐face visits reduced risk of cardiovascular death (OR 0.624 [95% CI 0.597–0.651]).

**Conclusions:**

Guideline‐directed medical therapy implementation and follow‐up after HF hospitalization was achievable in ~400 000 patients and was associated with cardiovascular benefits 1‐year post‐initiation.

## Introduction

Affecting more than 64 million people worldwide, heart failure (HF) has been recognized as a significant clinical and public health problem associated with lifelong impaired quality of life, substantial healthcare expenditure and strikingly high mortality, particularly among those aged 65 years and older.^1^ In a systematic meta‐analysis of 1.5 million individuals hospitalized for HF, 13.2% were readmitted within 30 days and 35.7% within 1 year; 7.6% died within 30 days and 23.3% died within 1 year.^2^ Despite the availability of many effective therapies for HF, implementation of these therapies remains a global challenge, with only a small percentage of patients with HF receiving optimal guideline‐directed medical therapy (GDMT) due to a lack of awareness and/or a lack of training on the guidelines.^3^, ^4^ In the Canadian Heart Failure study, 75% of people with HF were eligible for GDMT; however, only 9% were discharged with treatment after HF hospitalization.^5^ Similarly, in the Change the Management of Patients with Heart Failure study of 3518 patients in the United States, 86% of eligible patients were not prescribed angiotensin receptor–neprilysin inhibitors (ARNI), and only 1% received target doses of optimal GDMT.^4^, ^5^ The STRONG‐HF trial was the first to assess the impact of rapid up‐titration of GDMT therapies in patients with acute HF; results showed that patients who were prescribed more GDMT had lower rates of readmission for HF and lower mortality rates at 6 months.^6^, ^7^ The European Society of Cardiology recommends initiating optimal GDMT for acute HF before discharge and during frequent follow‐up visits to reduce the risk of readmission or death.^8^ However, the feasibility and/or benefits of applying optimal GDMT recommendations for HF at a nationwide level remains to be determined.

China has approximately 12.1 million patients living with HF, accounting for more than 10% of patients with HF worldwide, with annual HF‐related medical costs exceeding 250 billion Yuan (US$31 billion).^9^ Chinese HF guidelines were developed in 2007 and updated in 2014 and 2018.^10^, ^11^, ^12^, ^13^ However, implementing these HF guidelines is challenging, as nearly 50% of patients with HF in China live in rural areas^14^ where they may only have access to secondary (non‐specialized in HF) centres which have a lack of awareness of guidelines and training, resulting in poorer quality care compared with tertiary (specialized HF) centres.^15^ As a result, the Committee on Heart Failure launched the National Heart Failure Center Accreditation Program (HF‐CAP),^16^ which aimed to improve implementation of optimal GDMT for HF, perform quality control analyses quarterly, identify problems in HF management in both secondary and tertiary hospitals and in all regions, propose targeted solutions to guide quality improvement, and educate patients and relatives through clinic visits and/or phone calls to improve adherence to treatment.

This prospective study of the HF‐CAP in China aimed to (i) describe the association between implementing a nationwide GDMT programme in hospitalized patients with HF and hospital readmission and/or mortality rates; (ii) describe the association between time since centre accreditation and these outcomes; and (iii) explore the potential association of post‐discharge visits or phone calls on patient outcomes.

## Methods

### Study design and population

We used a longitudinal, prospective, pre‐post comparison design in hospitals that successfully achieved HF‐CAP accreditation. The HF‐CAP organizational framework and regional HF care network are shown in online supplementary *Figures* *S1* and *S2*. HF‐CAP is an ongoing, prospective quality improvement programme launched on 1 January 2017, designed to implement optimal HF therapies in China. All hospitals registered in China were invited to apply for HF‐CAP membership. To be accredited, the minimum number of patients with HF admitted per year in secondary and tertiary centres was >100 and >300, respectively. Detailed accreditation criteria are presented in online supplementary *Table* *S1*. After accreditation, the HF‐CAP protocols include intensive monitoring and evaluation procedures to ensure that accreditation requirements continue to be met, that is, routine monthly audits and feedback, externally facilitated quality improvement workshops, opportunities for national/regional awards with or without financial incentives, and patient education sessions. Further, HF‐CAP centres are re‐accredited through unannounced on‐site inspections by Committee on Heart Failure assessors every 3 years to ensure they maintain high standards and compliance with best practices. The processes of accreditation, maintaining accreditation and core components of continuous monitoring post‐certification (including responsibilities of HF centres, training programmes and patient education) are shown in online supplementary *Figure* *S3* and *Tables* *S2*–*S4*.

After accreditation had been awarded, each centre was required to record the data of patients with HF who had an unplanned hospital admission in the 6–12 months prior to formal accreditation and all patients who were admitted after accreditation. Recorded data included demographics, medical history, clinical information, treatment at discharge, length of hospital stay, dates of visits and phone calls, detailed treatment and 1‐year outcomes.

To evaluate the association between HF‐CAP and patient outcomes, patients were categorized into four groups according to the time between hospital admission and centre accreditation: (1) 6–12 months before centre accreditation (Pre‐accreditation); (2) >0 to ≤12 months after accreditation (Year 1 or Y1); (3) >12 to ≤24 months after accreditation (Year 2 or Y2); and (4) >24 months after accreditation (Year 2+ or Y2+).

### Data collection

All accredited centres were responsible for collecting and reporting their own data for consecutively hospitalized patients with HF to the HF‐CAP database. The diagnosis of HF was made by hospital physicians according to the national HF guidelines and the type of HF was classified via left ventricular ejection fraction (LVEF) from echocardiography at the index hospitalization, confirmed for the final adjudication by a certified cardiologist. Baseline data were collected from medical records during the index hospitalization. After discharge, patients were followed up by phone calls after 7 days (with an additional 7‐day flexibility), then by outpatient visit in the first month (± 14 days), the third month (± 30 days) and the first year (± 90 days). During follow‐up, patients may have undergone echocardiography and/or laboratory tests. Patients who missed their outpatient appointment received a follow‐up phone call.

The data coordination team was responsible for the daily monitoring of the database. The data items included in the HF‐CAP registry are listed in online supplementary *Table* *S5*. The Committee on Heart Failure reviewed the data recorded by HF‐CAP centres annually.

Ethical approval for the HF‐CAP was obtained from the institutional review boards of the Central Ethics Committee of Beijing Hospital (2018BJYYEC‐059‐02). Informed consent was obtained from the registered hospitals and research approval was obtained for data collection in the programme. The current study was further approved by the institutional review board of Zhongshan Hospital, Fudan University, for the use of deidentified data (Approval No. B2024‐038). This study followed the Strengthening the Reporting of Observational studies in Epidemiology reporting guideline^17^ and is registered with the Chinese Clinical Trials Registry (chictr.org.cn; identifier: ChiCTR1800017226).

### Study endpoints

The primary endpoint was the 1‐year composite event rate of the first unscheduled readmission due to worsening HF or cardiovascular (CV) death. Events were identified during scheduled outpatient clinic visits and/or phone call follow‐ups; if a patient did not attend a scheduled visit, the patient and/or next of kin was contacted to confirm if HF or CV death had occurred. Further, a surveillance system was in place to identify unplanned HF hospitalizations in all HF‐CAP hospitals. In each centre, the diagnosis of readmission for HF or CV death was reviewed by a panel of physicians and then verified by a CV specialist for final confirmation; death of an unknown cause was considered to be of non‐CV aetiology. The following covariates were used: age, sex, comorbidities, LVEF, type of hospital (secondary or tertiary) and GDMT where appropriate.

Secondary endpoints were 1‐year HF readmission alone, 1‐year CV death alone and the association between scheduled phone calls or clinic visits and outcomes.

### Statistical analyses

Summary statistics (mean, standard deviation [SD], median, interquartile range [IQR] and proportion) were used to characterize the study population. The Pearson *χ* ^2^ ANOVA or Kruskal–Wallis tests were used to compare categorical and continuous variables as appropriate. *P*‐values for the trend were calculated using the Cochran–Armitage trend test. Logistic regression models, adjusted for potential confounders, were used to examine the association between the time from hospital admission to centre accreditation and outcomes. Statistical models were evaluated for goodness‐of‐fit using the likelihood ratio test. Hierarchical logistic regression modelling adjusted for random cluster was used to manage potential hospital cluster effects and nested sources of variability in the two hospital types. Interactions between time since accreditation (pre‐accreditation, post‐accreditation [at Y1, Y2, Y2+]) and sex (male or female) were included in the models to analyse the association of HF‐CAP with accreditation status or sex differences with the outcomes. For sensitivity analyses, an inverse probability of treatment weighting outcome model was used to eliminate differences in baseline characteristics of patients in the pre‐ and post‐accreditation [at Y1, Y2, Y2+] groups. Moreover, sensitivity analyses were used to examine the robustness of the result by performing the analyses under a range of plausible assumptions about the multivariate logistic models. All analyses were performed using SAS 9.4 statistical software (SAS Institute, Cary, NC, USA) and R software version 4.3.2 (R Foundation for Statistical Computing, Vienna, Austria). All *p*‐values are two‐sided; *p* < 0.05 was considered statistically significant.

## Results

### Baseline characteristics

**Figure 1:**
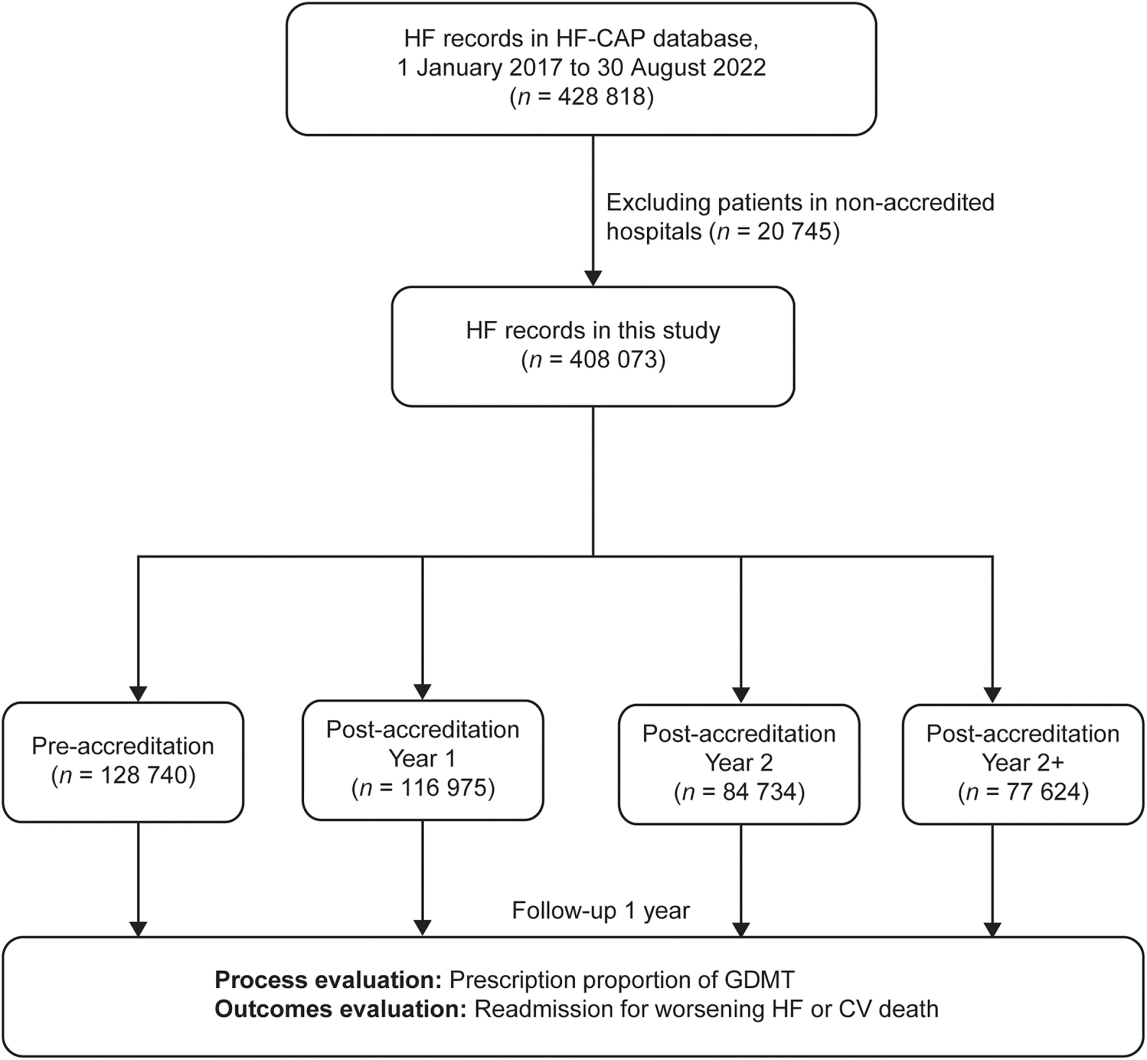
Study population selection and follow‐up. Heart failure (HF) patients were divided into four groups based on the time since the centre was accredited. Pre‐accreditation (Pre‐): 6–12 months before accreditation; Post‐accreditation Year 1: >0 and ≤12 months after accreditation; Post‐accreditation Year 2: >12 and ≤24 months after accreditation; Post‐accreditation Year 2+: >24 months after accreditation. A total of 21 948 (5.38%) patients were lost to follow‐up 1 year after discharge. CV, cardiovascular; GDMT, guideline‐directed medical therapy; HF‐CAP, National Heart Failure Center Accreditation Program.

The data for this study were obtained from the HF‐CAP database. We identified 428 818 HF admissions from 1 January 2017 to 30 August 2022. We excluded 20 745 patients from 74 hospitals that had not successfully been accredited by HF‐CAP by 30 August 2022. Thus, the final sample size for this study was 408 073 patients admitted to hospital for HF, with a total of 21 948 (5.38%) patients lost to follow‐up 1 year after discharge. The geographical locations of the centres with patients included in the study are displayed in online supplementary *Figure* *S4* and *Figure* *1* shows the study population selection.

From the 646 centres participating in HF‐CAP, 128 740 patients with HF were recorded prior to the accreditation (6–12 months before accreditation) of their centres (Pre‐accreditation group); the remaining patients were separated into Y1, Y2 and Y2+ groups as appropriate (*Figure* *1*). Baseline patient characteristics by time since accreditation are presented in *Table* *1*. Overall, the mean (SD) age was 69.5 (13.3) years and 41.1% were female. Patients in the Y2+ group were more likely to have a history of percutaneous coronary intervention (*p* < 0.001) and to have echocardiography performed at index hospitalization (*p* < 0.001). All other baseline characteristics were similar across groups.

**Table 1:** Baseline characteristics of patients hospitalized with heart failure by time since centre accreditation

Characteristic	All patients (*n* = 408 073)	Pre‐accreditation (*n* = 128 740)	Post‐accreditation		
			Year 1 (*n* = 116 975)	Year 2 (*n* = 84 734)	Year 2+ (*n* = 77 624)
Demographic					
Female sex, *n* (%)	167 722 (41.1)	55 018 (42.7)	48 442 (41.4)	34 178 (40.3)	30 084 (38.8)
Age (years), mean ± SD	69.5 ± 13.3	70.7 ± 13.0	69.4 ± 13.2	69.0 ± 13.5	68.4 ± 13.7
Median (IQR)	71 (62–79)	72 (63–80)	71 (62–79)	70 (61–79)	70 (60–78)
History, comorbidities, *n* (%)					
Hypertension	237 270 (58.1)	75 941 (59.0)	67 318 (57.6)	48 854 (57.7)	45 157 (58.2)
Type 2 diabetes mellitus	111 582 (27.3)	34 646 (26.9)	31 258 (26.7)	23 451 (27.7)	22 227 (28.6)
Hyperlipidaemia	58 814 (14.4)	21 212 (16.5)	16 753 (14.3)	11 167 (13.2)	9682 (12.5)
Obesity	75 277 (18.5)	24 588 (19.1)	22 359 (19.1)	14 702 (17.4)	13 628 (17.6)
Ischaemic heart disease	250 504 (61.4)	81 179 (63.1)	71 966 (61.5)	51 055 (60.3)	46 304 (59.7)
Prior myocardial infarction	93 778 (23.0)	28 952 (22.5)	26 788 (22.9)	19 769 (23.3)	18 269 (23.5)
Atrial fibrillation	134 715 (33.0)	44 211 (34.3)	39 320 (33.6)	27 380 (32.3)	23 804 (30.7)
Stroke	49 847 (12.2)	16 166 (12.6)	14 333 (12.3)	10 412 (12.3)	8936 (11.5)
Renal insufficiency	51 042 (12.5)	17 392 (13.5)	13 702 (11.7)	10 065 (11.9)	9883 (12.7)
COPD or asthma	39 077 (9.6)	14 961 (11.6)	11 229 (9.6)	6914 (8.2)	5973 (7.7)
Depression/anxiety	5565 (1.4)	1943 (1.5)	1441 (1.2)	914 (1.1)	1267 (1.6)
Anaemia	86 433 (21.2)	29 071 (22.6)	24 260 (20.7)	17 494 (20.7)	15 608 (20.1)
History, device/procedure, *n* (%)					
PCI	84 667 (20.8)	17 720 (13.8)	22 259 (19.0)	19 643 (23.2)	25 045 (32.3)
CABG	3160 (0.8)	1115 (0.9)	987 (0.8)	643 (0.8)	415 (0.5)
Pacemaker	14 151 (3.5)	4805 (3.7)	3964 (3.4)	2832 (3.3)	2550 (3.3)
ICD	3439 (0.8)	944 (0.7)	919 (0.8)	794 (0.9)	782 (1.0)
CRT‐P	1206 (0.3)	382 (0.3)	363 (0.3)	251 (0.3)	210 (0.3)
CRT‐D	3144 (0.8)	993 (0.8)	889 (0.8)	578 (0.7)	684 (0.9)
Vitals on admission, mean ± SD					
BMI (kg/m^2^)	23.6 ± 3.6	23.5 ± 3.6	23.6 ± 3.6	23.7 ± 3.7	23.8 ± 3.6
SBP (mmHg)	131.2 ± 24.3	132.1 ± 24.3	131.1 ± 24.3	129.1 ± 23.9	128.4 ± 23.0
DBP (mmHg)	77.5 ± 14.1	77.6 ± 14.0	77.8 ± 14.2	76.5 ± 14.3	75.7 ± 13.5
Heart rate (bpm)	83.1 ± 20.5	83.8 ± 20.9	83.7 ± 20.7	82.5 ± 20.0	77.8 ± 17.6
Laboratory on admission					
NT‐proBNP					
Patients assessed, *n* (%)^a^	317 404 (77.8)	99 574 (77.3)	94 822 (81.1)	66 374 (78.3)	56 634 (73.0)
Value (pg/ml), median (IQR)	2252 (903–5530)	2560 (1000–6323)	2318 (937–5665)	2062 (846–4981)	1905 (798–4625)
LVEF					
Patients assessed, *n* (%)	362 400 (88.8)	108 223 (84.1)	106 294 (90.9)	76 406 (90.2)	71 477 (92.1)
LVEF (%), mean ± SD	46.6 ± 13.7	48.0 ± 13.8	46.5 ± 13.6	46.0 ± 13.6	45.4 ± 13.7
NYHA class on admission, *n* (%)					
I	5706 (1.4)	1252 (1.0)	1235 (1.1)	1455 (1.7)	1764 (2.3)
II	43 182 (10.6)	9919 (7.7)	10 249 (8.8)	9543 (11.3)	13 471 (17.4)
III	110 929 (27.2)	25 335 (19.7)	27 065 (23.1)	26 082 (30.8)	32 447 (41.8)
IV	76 250 (18.7)	20 199 (15.7)	18 281 (15.6)	17 412 (20.6)	20 358 (26.2)
Missing	172 006 (42.2)	72 035 (56.0)	60 145 (51.4)	30 242 (35.7)	9584 (12.4)
HF type, *n* (%)					
HFrEF	131 083 (32.1)	35 207 (27.4)	38 713 (33.1)	28 745 (33.9)	28 418 (36.6)
HFmrEF	74 101 (18.2)	21 510 (16.7)	22 000 (18.8)	15 673 (18.5)	14 918 (19.2)
HFpEF	157 216 (38.5)	51 506 (40.0)	45 581 (39.0)	31 988 (37.8)	28 141 (36.3)
HF unclassifiable^b^	45 673 (11.2)	20 517 (15.9)	10 681 (9.1)	8328 (9.8)	6147 (7.9)
Admission year, *n* (%)					
2017	13 145 (3.2)	11 956 (9.3)	1189 (1.0)	0 (0.0)	0 (0.0)
2018	53 736 (13.2)	28 440 (22.1)	23 943 (20.5)	1353 (1.6)	0 (0.0)
2019	91 458 (22.4)	34 528 (26.8)	32 458 (27.8)	24 472 (28.9)	0 (0.0)
2020	90 635 (22.2)	28 475 (22.1)	24 555 (21.0)	23 719 (28.0)	13 886 (17.9)
2021	85 132 (20.9)	20 930 (16.3)	22 002 (18.8)	16 823 (19.9)	25 377 (32.7)
2022	73 967 (18.1)	4411 (3.4)	12 828 (11.0)	18 367 (21.7)	38 361 (49.4)
Accreditation type, *n* (%)					
Tertiary hospitals	336 322 (82.4)	103 542 (80.4)	93 424 (79.9)	69 968 (82.6)	69 388 (89.4)
Secondary hospitals	71 751 (17.6)	25 198 (19.6)	23 551 (20.1)	14 766 (17.4)	8236 (10.6)

Time since accreditation groups: Pre‐accreditation: 6–12 months before accreditation; Post‐accreditation Year 1: >0 and ≤ 12 months after accreditation; Post‐accreditation Year 2: >12 and ≤ 24 months after accreditation; Post‐accreditation Year 2+: >24 months after accreditation.

BMI, body mass index; CABG, coronary artery bypass graft; COPD, chronic obstructive pulmonary disease; CRT‐D, cardiac resynchronization therapy with defibrillator; CRT‐P, cardiac resynchronization therapy with pacemaker; DBP, diastolic blood pressure; HF, heart failure; HFmrEF, heart failure with mildly reduced ejection fraction; HFpEF, heart failure with preserved ejection fraction; HFrEF, heart failure with reduced ejection fraction; ICD, implantable cardioverter‐defibrillator; IQR, interquartile range; LVEF, left ventricular ejection fraction; NT‐proBNP, *N*‐terminal pro‐B‐type natriuretic peptide; NYHA, New York Heart Association; PCI, percutaneous coronary intervention; SBP, systolic blood pressure; SD, standard deviation.

^a^ Number of patients (%) with NT‐proBNP concentrations at index hospitalization.

^b^ Missing baseline LVEF.

The rates of in‐hospital mortality were lower in the post‐accreditation Y2+ group (4.3%) than the Y1 (5.6%), Y2 (5.5%) and Pre‐accreditation groups (5.6%) (*p* for trend <0.001). The median (IQR) length of hospital stay for all patients was 8 (6–12) days, with no significant differences found in the length of stay among groups. Patient characteristics in tertiary HF centres versus secondary HF centres are presented in online supplementary *Table* *S6*.

### Proportions of patients prescribed guideline‐directed medical therapy before and after centre accreditation

**Figure 2:**
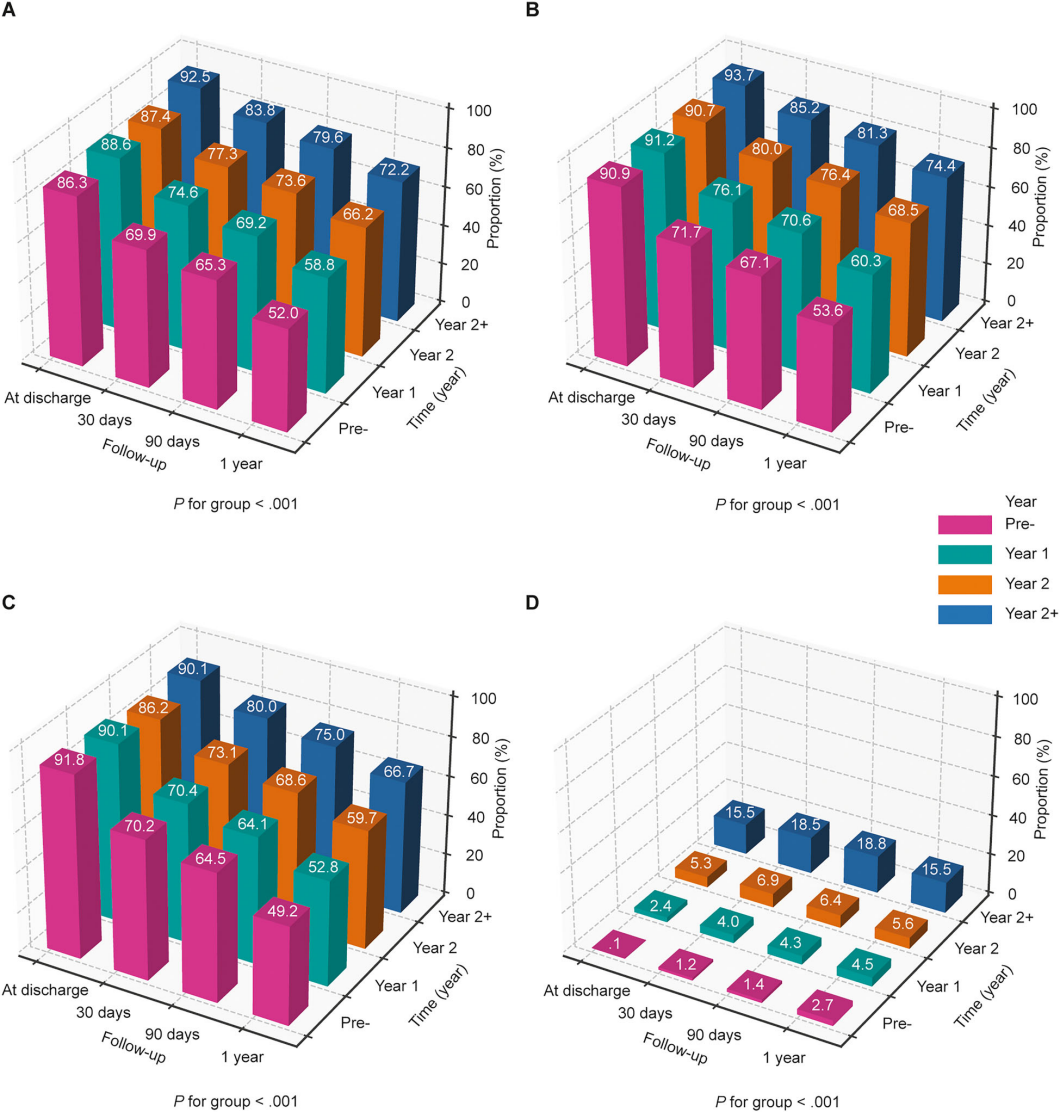
Prescription rates of (*A*) angiotensin‐converting enzyme inhibitors/angiotensin receptor blockers/angiotensin receptor–neprilysin inhibitors (ACEi/ARB/ARNI), (*B*) beta‐blockers, (*C*) mineralocorticoid receptor antagonists, and (*D*) sodium–glucose co‐transporter 2 inhibitors. Data shown are in patients without contraindications from discharge to 1 year after discharge in the National Heart Failure Center Accreditation Program centres divided into four groups: before (Pre‐accreditation [Pre‐]), and 1 (Year 1), 2 (Year 2) or more than 2 years (Year 2+) after accreditation. Increases in ACEi/ARB/ARNI prescription rates in Year 1, Year 2 and Year 2+ groups compared with Pre‐ are related to striking increases in the use of ARNI (online supplementary *Figure* *S5*).

Comparisons of the proportion of patients prescribed angiotensin‐converting enzyme inhibitors (ACEi)/angiotensin receptor blockers (ARB)/ARNI, beta‐blockers, mineralocorticoid receptor antagonists (MRA) and sodium–glucose co‐transporter 2 inhibitors (SGLT2i) in the four groups in patients with HF, excluding contraindications, based on time since centre accreditation, are shown in *Figure* *2* and online supplementary *Table* *S7*, which are further split by HF type in online supplementary *Table* *S8*. The rates of ACEi/ARB/ARNI, beta‐blockers, and MRA (but not SGLT2i) prescribed in patients decreased from hospital discharge to 1‐year follow‐up in the four studied groups (Pre‐accreditation, Y1, Y2, Y2+). In the Pre‐accreditation group, ACEi/ARB/ARNI, beta‐blockers and MRA were all prescribed in 86.3%, 90.9% and 91.8% of patients with HF at discharge, respectively, which decreased to 52.0%, 53.6% and 49.2% of patients 1 year later (*p* for trend <0.001). In the Y1, Y2 and Y2+ groups, prescription of those three classes of HF medications incrementally increased. The best results were observed for centres accredited for more than 2 years (Y2+), with more than 90% of patients prescribed ACEi/ARB/ARNI, beta‐blockers and MRA at discharge, and >65% after 1 year. For ACEi/ARB/ARNI prescriptions, the rate from discharge to 1 year later was incrementally better at −34.3%, −29.8%, −21.2% and −20.3%, in the Pre‐accreditation, Y1, Y2 and Y2+ groups, respectively, indicating that the longer the centres had been accredited, the better the prescription of the four pillars of HF therapy and the smaller the decrease in the year following discharge (*p* for trend <0.001). Similar trends were also seen for the prescription of beta‐blockers (*p* for trend <0.001) and MRA (*p* for trend <0.001). Online supplementary *Figure* *S5* shows that increases in the proportion of patients prescribed ACEi/ARB/ARNI from discharge to 1 year after discharge was mostly due to an increased prescription of ARNI (*p* for trend <0.001). As indicated in online supplementary *Table* *S8*, prescription of SGLT2i increased overall from approximately 0% in the Pre‐accreditation group at discharge, to more than 15% in the Y2+ group at discharge (there was no significant change between discharge and 1 year later).

When looking at patients by LVEF type, the prescription rates of ACEi/ARB/ARNI, beta‐blockers, MRA and SGLT2i were slightly lower in patients with HF with mildly reduced or preserved ejection fraction (HFmrEF/HFpEF) compared with patients with HF with reduced ejection fraction (HFrEF), though trends within these groups according to time since accreditation were similar to those described above (online supplementary *Table* *S8*).

### Rates of telephone/clinical follow‐up visits before and after centre accreditation

The proportion of patients who attended more than two scheduled phone call follow‐ups in the year following discharge incrementally increased with time since accreditation from 40.5% in the Pre‐accreditation group to 61.8% in the Y2+ group; the number of scheduled face‐to‐face clinic visits also incrementally increased with time since accreditation from 12.0% to 19.7% in Pre‐accreditation and Y2+ groups (*p* for trends <0.001 for all). The frequency of phone calls versus face‐to‐face clinic follow‐up visits can be found in online supplementary *Figure* *S6* and *Table* *S9*.

### Outcomes of readmission for worsening heart failure and/or cardiovascular death

In the Pre‐accreditation group (*n* = 128 740), 19 380 patients had an event relating to the primary endpoint in the first year of follow‐up; 13 456 patients had their first episode of readmission for worsening HF; and 8908 experienced CV death (online supplementary *Table* *S10*). In the Pre‐accreditation group, this corresponds to an annual rate (95% CI) of HF readmission and/or CV death of 15.1% (14.9–15.3). Compared with the Pre‐accreditation group, the annual incidence rate of the primary endpoint decreased with increasing time since centre accreditation (13.1% [12.9–13.3] in the Y1 group, 12.5% [12.3–12.8] in the Y2 group, and 10.7% [10.5–10.9] in the Y2+ group; *p* for trend <0.001).

The trend observed in the overall composite primary endpoint was also apparent when looking at the two components of the primary endpoint (readmission for worsening HF and CV death) as separate factors. The annual rate of readmission for worsening HF was 10.5% (10.3–10.6) in the Pre‐accreditation group, 8.9% (8.7–9.0) in the Y1 group, 8.7% (8.5–8.9) in the Y2 group, and 6.8% (6.6–7.0) in the Y2+ group (*p* for trend <0.001). The rate of 1‐year CV death alone was 6.9% (6.8–7.1) in the Pre‐accreditation group, 6.3% (6.1–6.4) in the Y1 group, 6.0% (5.8–6.1) in the Y2 group, and 5.3% (5.1–5.4) in the Y2+ group (*p* for trend <0.001). The trends of the annual rates for the primary endpoint were also observed by group and by clinically relevant subgroups (sex, HF type, hospital expertise in HF) (online supplementary *Table* *S11*).

**Figure 3:**
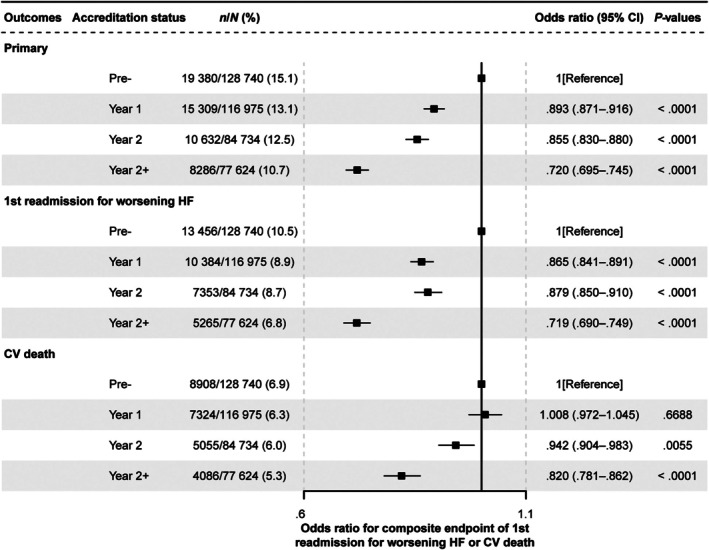
Odds ratios (95% confidence interval [CI]) of 1‐year composite endpoint. Composite endpoint: readmissions for worsening heart failure (HF) or cardiovascular (CV) death in patients hospitalized with HF divided into four groups: before (Pre‐accreditation [Pre‐]), and 1 (Year 1), 2 (Year 2) or more than 2 years (Year 2+) after accreditation of the centre by the National Heart Failure Center Accreditation Program. Adjusted for age, sex, region, admission year, left ventricular ejection fraction, log transformation for N‐terminal pro‐B‐type natriuretic peptide, comorbid conditions (prior myocardial infarction, diabetes, hypertension, atrial fibrillation, renal insufficiency, chronic obstructive pulmonary disease, anaemia), angiotensin‐converting enzyme inhibitors/angiotensin receptor blockers/angiotensin receptor–neprilysin inhibitors, beta‐blockers and mineralocorticoid receptor antagonists, as appropriate.

**Figure 4:**
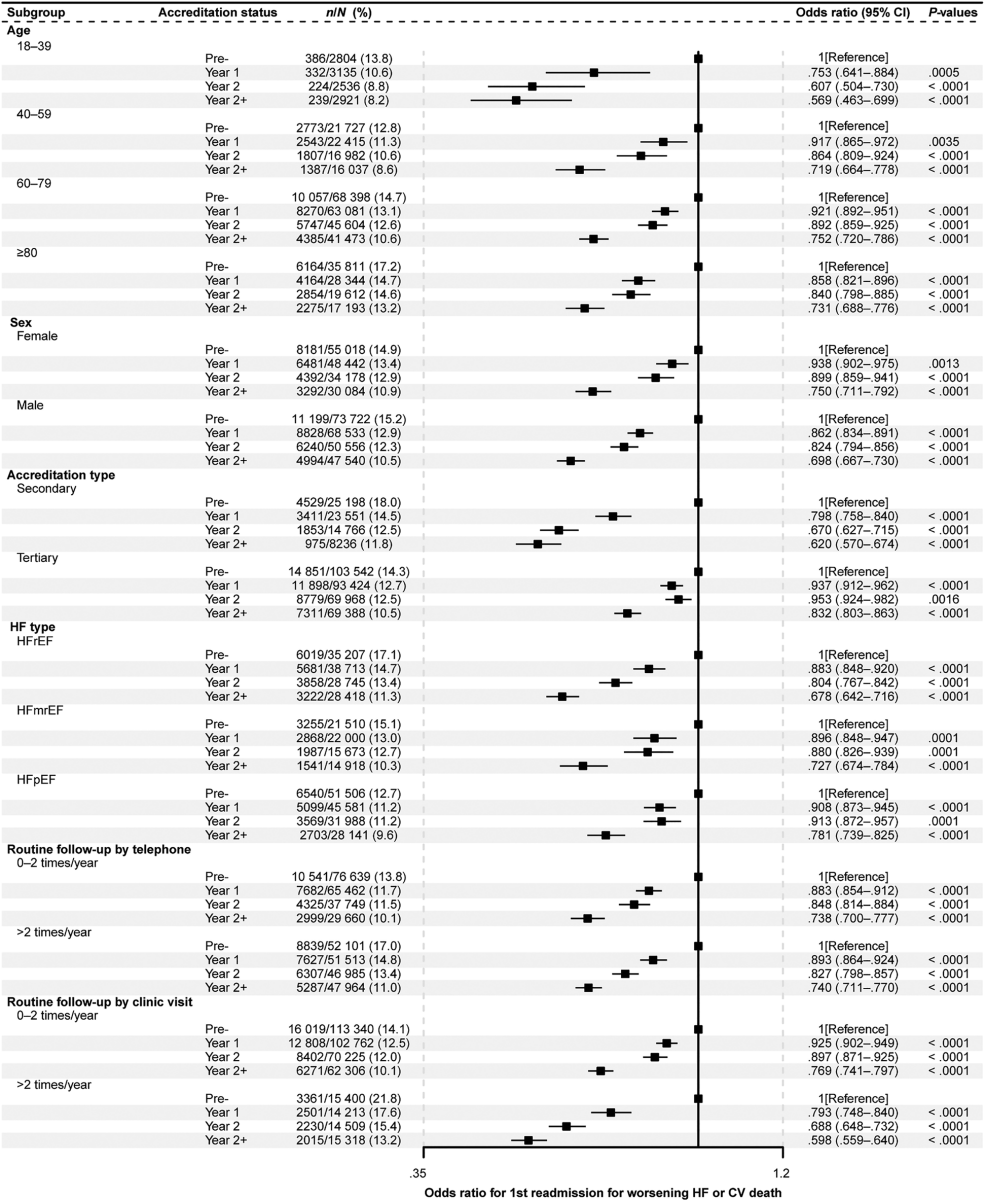
One‐year composite endpoint by clinically relevant subgroups. Composite endpoint: readmissions for worsening heart failure (HF) or cardiovascular death in patients hospitalized with HF before (Pre‐), and 1 (Year 1), 2 (Year 2), or more than 2 years (Year 2+) after accreditation of the centre by the National Heart Failure Center Accreditation Program. CI, confidence interval; HFmrEF, heart failure with mildly reduced ejection fraction; HFpEF, heart failure with preserved ejection fraction; HFrEF, heart failure with reduced ejection fraction.

The odds of readmission for worsening HF or CV death decreased with increasing time since centre accreditation. Multivariate analysis adjusting for potential confounders indicated that, compared with the Pre‐accreditation group, the odds ratios (OR) and 95% confidence intervals (CIs) of the risk of 1‐year CV death and/or HF readmission were 0.893 (0.871–0.916), 0.855 (0.830–0.880), and 0.720 (0.695–0.745) in the Y1, Y2 and Y2+ groups, respectively (*Figure* *3*). Robustness of the main analysis was confirmed through sensitivity analyses using inverse probability of treatment weighting (online supplementary *Table* *S12*); after adjustment for percutaneous coronary intervention, coronary artery bypass grafting, and hospital level (tertiary vs. secondary; online supplementary *Table* *S13*); and when patients without echocardiography were excluded (online supplementary *Table* *S14*). Similar results were seen when looking at the two components of the primary endpoint as separate factors: the risk of 1‐year HF readmission had an OR (95% CI) of 0.865 (0.841–0.891), 0.879 (0.850–0.910) and 0.719 (0.690–0.749) in the Y1, Y2 and Y2+ groups, respectively. Regarding risk of CV death, OR (95% CI) was 1.008 (0.972–1.045) for Y1, 0.942 (0.904–0.983) for Y2, and 0.820 (0.781–0.862) for Y2+ groups (*Figure* *3*). The OR for the primary endpoint decreased with increasing time since centre accreditation in almost all subgroups and was significantly lower in all subgroups versus the Pre‐accreditation group (*Figure* *4* and online supplementary *Figures* *S7* and *S8*). Reduced OR for the primary endpoint was seen in HF patients hospitalized in secondary or tertiary centres and in patients with HFrEF, HFmrEF or HFpEF.

### Association of primary outcome with telephone/clinical follow‐up visits

**Figure 5:**
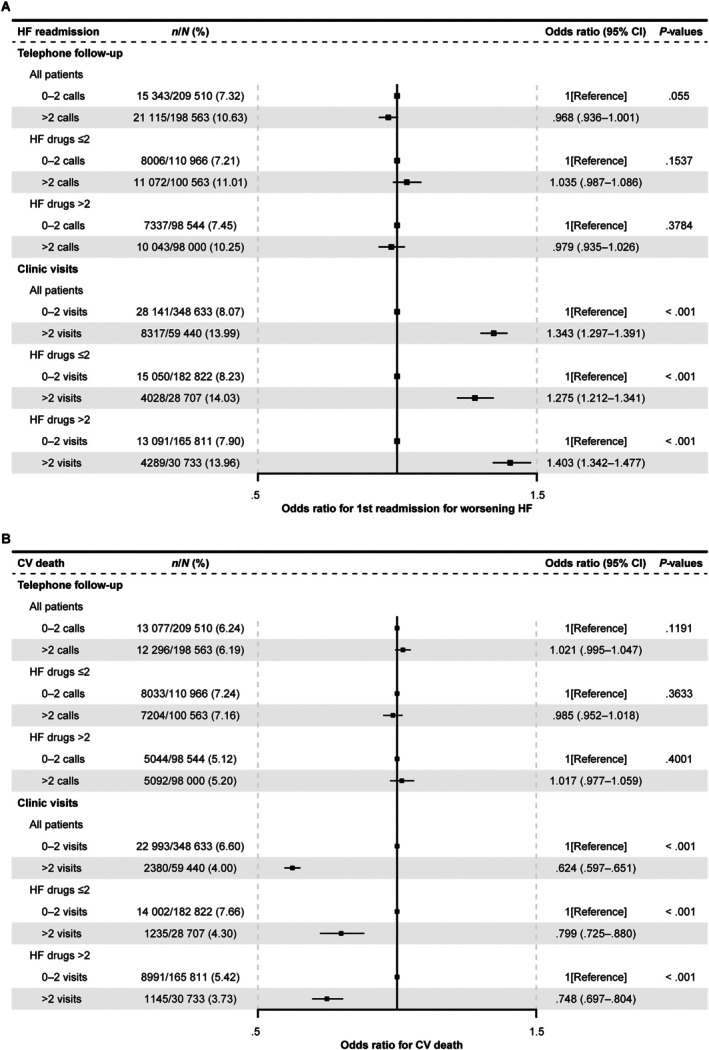
Odds ratios of telephone/clinic follow‐up visits by 1‐year secondary endpoints. Odds ratios (95% confidence intervals [CI]) of association between number of phone call or face‐to‐face follow‐ups and number of heart failure (HF) drugs received with (*A*) worsening HF and (*B*) 1‐year cardiovascular (CV) death.

The association between outcomes and scheduled phone calls and/or face‐to‐face visits (*Figure* *5*) shows that the OR for a primary endpoint event was similar between subgroups with low or high rates of phone calls and face‐to‐face clinic visits. *Figure* *5* further shows that a high rate of phone calls was associated with no change in CV death nor in HF readmission, while a high rate of clinic visits was associated with lower risk of CV death but higher risk of HF readmission. The association between HF‐CAP and patient outcomes, number of phone calls and/or face‐to‐face visits, and number of HF drugs prescribed to patients shows that the OR for a primary endpoint event was not significant based on the number of drugs prescribed at the phone call follow‐ups. However, the OR was significantly different for those prescribed ≤2 HF drugs with a high rate of clinic visits versus low rate of clinic visits. Of note, no difference was seen in the main characteristics among patients receiving high or low rates of phone calls or clinic visits (online supplementary *Table* *S15*).

## Discussion

In this prospective study, we showed that implementing a nationwide, multi‐annual programme of optimal GDMT in over 400 000 patients with HF across China successfully achieved a rapid and significant increase in the proportion of patients who were prescribed HF therapies, and a subsequent decrease in readmissions and/or CV death. There was a parallel reduction in CV risks within the entire study population of hospitalized patients with HF, including in all subgroups examined (*Graphical Abstract*). These data from almost all regions of China suggest that this concept could be applied globally to improve quality of life and survival in patients with HF within 1 year.

Implementation of optimal GDMT in patients with HFrEF is recommended based on data showing that the combination of ACEi/ARB/ARNI, beta‐blockers, MRA and recently SGLT2i (the four pillars of HF therapy) is associated with best outcomes.^18^ The STRONG‐HF trial further demonstrated that prescription of the combination of ACEi/ARB/ARNI, beta‐blockers and MRA within a few weeks of hospital discharge is safe and associated with marked improvement in 6‐month CV death and/or HF readmission rates in all patients with HF, regardless of LVEF category.^6^ In STRONG‐HF, safety was assessed by post‐discharge face‐to‐face visits that included clinical and biological examinations.^6^, ^7^ The present study is the first to prospectively assess and conclude that implementation of optimal GDMT for HF at a large scale (more than 600 secondary and tertiary centres and >400 000 patients with HF) is feasible. Further, optimal GDMT implementation under the HF‐CAP was associated with improved CV outcomes in hospitalized patients with HF, regardless of LVEF category, age, or sex. This study also showed that implementing an optimal GDMT programme in patients with HF was associated with benefits not only in tertiary centres, as seen in STRONG‐HF, but also in secondary centres. It should be noted that whilst the prescription rates of ACEi/ARB/ARNI, beta‐blockers, MRA, and SGLT2i were slightly lower in patients with HFpEF or HFmrEF compared with those who had HFrEF, there is no evidence to support the efficacy of ACEi/ARB or beta‐blockers in patients with HFpEF. Therefore, these medications would be expected to be prescribed less in these patients, which would explain the reduced prescription rates. Overall, the benefits outlined in this study have the potential to be applied globally in any ‘accredited’ hospital (even those not specialized in HF). In addition, although the STRONG‐HF trial and the present HF‐CAP analysis were conducted in patients with acute HF, there may be potential to apply this concept more broadly in CV disease to further improve patient outcomes.

The estimated total number of people aged ≥25 years with HF in China is 12.1 million (95% CI 10.9–13.2).^19^ Our study showed that compared with Pre‐accreditation, patients admitted to hospital 2 years or more after accreditation had a markedly decreased annual CV disease mortality (6.9% [95% CI 6.8–7.1] vs. 5.3% [95% CI 5.1–5.4]) as a result of the HF‐CAP; if HF‐CAP was extended to the whole country, 193 600 (95% CI 174 400–211 200) HF‐related deaths could be avoided annually. Conversely, if HF management was implemented without a GDMT programme, HF‐related premature mortality in China may continuously increase.^20^

The HF‐CAP included close monitoring of the centres to achieve safe and effective implementation of HF therapies in all patients with HF. Phone call/outpatient clinic follow‐up visits were one of the indicators used to assess quality management and were a key component of the certification programme. In the present study, the proportion of phone calls and clinic visits rose from Pre‐accreditation to Y2+, and it is reasonable to assume that this increase is directly attributable to the accreditation process. In addition, a higher number of clinic visits was associated with increased hospitalization (close observation likely identified any health issues requiring readmission); this was, in turn, associated with decreased mortality. This close monitoring during readmission also suggests patients are more likely to receive necessary changes to medication, which may also increase survival. Phone call visits did not follow this trend and were not associated with increased survival, which could potentially be explained by the more in‐depth assessment which occurs at face‐to‐face clinic visits. Future studies should examine the impact of the number of visits on patient outcomes in more detail.

The HF‐CAP was applied in selected accredited centres. Various processes are used to assess if an institution meets the accreditation criteria. The process of accreditation can be voluntary or mandatory.^21^, ^22^, ^23^ We used the Chinese Heart Association‐defined accreditation implementation standards,^16^ and the National Chest Pain Centers Program (NCPCP) accreditation findings with the clinical benefits in the NCPCP population as reference.^24^ A main finding of our study is the rapid improvement in CV benefits within 1 year of implementation of HF‐CAP followed by an incremental improvement in outcome in the two following years. This parallels the increased proportion of patients treated with GDMT within 1 year of HF‐CAP followed by a further increase in the following years. This incremental increase seen in the years following accreditation could be for several reasons. One potential explanation is the comprehensive, structured HF‐CAP, which included regular monitoring to ensure sites were following the responsibilities that come with being an accredited site; another explanation is that the proportion of patients receiving optimal treatment increased over time and outcomes followed. Accredited sites must ensure that all healthcare professionals are educated regularly; this would increase education across the site and may have improved outcomes over time. In addition, the more time since accreditation, the more likely it is that more patients were receiving optimal doses of treatment, with outcomes improving accordingly. The improvement in CV benefits and implementation of GDMT was observed in all patients with HF and subgroups, calling for a multi‐annual, global programme to achieve maximum improved CV outcomes.

This study included over 400 000 patients with all‐type HF and, to our knowledge, is currently one of the largest prospective cohort studies with many clinical characteristic variables available to extensively assess health issues related to HF. This study illustrates the benefits of introducing HF centre accreditation, providing insights for cardiologists, researchers, policymakers and stakeholders involved in the accreditation process. The HF‐CAP used standardized protocols and procedures and well‐established quality control measures to monitor and assure data quality, making the study results more clinically robust and suggesting potential application to other regions/countries. Use of pre‐specified inclusion criteria and minimal exclusions enabled us to increase the likelihood of identifying a broad and representative patient population.

The study has some limitations. Firstly, this study is a longitudinal analysis of associations, using a pre‐post design, which is less robust than randomized controlled trial design. Unlike randomized controlled trials, this quasi‐experimental research method cannot eliminate potential bias, meaning we were unable to fully investigate if it was implementing the HF‐CAP interventions, which lead to the improved outcomes. We addressed this limitation by conducting a sensitivity analysis using multiple statistical methods, all of which produced consistent conclusions. There may, however, still be unmeasured variables affecting the outcomes, such as variations in local practices or physician experiences. Another limitation is the lack of baseline outcome data from non‐participating hospitals. Prior to the programme, China had no nationwide HF registry. As part of the accreditation process, candidate hospitals were required to collect 6–12 months of pre‐implementation data, forming the Pre‐accreditation group (*n* = 128 740), which served as the control. After accreditation, data collection continued. However, we lack the information from hospitals that did not apply, which therefore limits generalizability and introduces potential selection bias.

Secondly, a cost‐effectiveness analysis of the accreditation was not carried out; therefore, while the approach was clinically effective, it is unknown whether the implementation of such a comprehensive programme is cost‐effective. Thirdly, the centres were followed for a limited number of years, and it is uncertain whether the benefits observed in this study may persist over time, with a potential risk of CV outcomes returning to pre‐accreditation levels. Fourthly, though patients predominantly managed via phone calls or clinic visits had similar baseline characteristics, differences in follow‐up management might have varied and we did not record clinical, biological, or imaging data during the scheduled visits. However, as described in the STRONG‐HF trial,^6^, ^7^ it is likely that parameters of congestion, for instance, improved in more optimally treated patients.^25^ Further studies should assess whether a GDMT implementation programme in a centre without accreditation yields similar results, or whether the accreditation process is needed to ensure continuous quality.

## Conclusion

In summary, our prospective study demonstrated that a nationwide, multi‐annual programme for implementing optimal GDMT under the HF‐CAP accreditation program in over 400 000 patients with HF in all regions of China resulted in a rapid and significant reduction in HF complications. The findings are a breakthrough for implementation science at a national level. These data also highlight the potential for this concept to be adopted globally for any HF patient, which may lead to improved quality of life and patient outcomes worldwide.

## Supporting information


**Appendix S1.** Supporting Information.

## Appendix

**Table:**

Center	Person in Charge
Beijing Hospital	Yang Jiefu
Zhongshan Hospital, Fudan University	Zhou Jingmin
The First Affiliated Hospital of Nanjing Medical University	Li Xinli
The First Affiliated Hospital, Sun Yat‐sen University	Dong Yugang
Guangdong Provincial People's Hospital	Li Liwen
The First Affiliated Hospital of Xi'an Jiaotong University	Bai Ling
The First Affiliated Hospital of Dalian Medical University	Liu Ying
Xinqiao Hospital, Army Medical University	Wang Jiang
Ruijin Hospital, Shanghai Jiao Tong University School of Medicine	Jin Wei
Chinese PLA General Hospital	Dong Wei
The Second Affiliated Hospital Zhejiang University School of Medicine	Xiang Meixiang
West China Hospital of Sichuan University	Zhang Qing
The Second Affiliated Hospital of Nanchang University	Li Ping
Xiamen Cardiovascular Hospital, Xiamen University	Dai Cuilian
The First Affiliated Hospital of Xinjiang Medical University	Aimann Mahmut

**Table:**

Center	Person in Charge
Beijing Chaoyang Hospital, Capital Medical University	Chen Mulei
Shanxi Cardiovascular Hospital	Han Huiyuan
Sichuan Provincial People's Hospital	Kong Hong
Anhui Provincial Hospital	Yan Ji
Tianjin Chest Hospital	Chen Shutao
Union Hospital, Tongji Medical College, Huazhong University of Science and Technology	Liao Yuhua
Fujian Provincial Hospital	Guo Yansong
Peking University First Hospital	Ma Wei
The Third Bethune Hospital of Jilin University	Yang Ping
Inner Mongolia People's Hospital	Zhao Xingsheng
Shandong Provincial Hospital	Yuan Haitao
The First Affiliated Hospital of Harbin Medical University	Li Weimin
The Second Affiliated Hospital of Harbin Medical University	Yu Bo
Wuhan Asian Heart Hospital	Su Xi
No. 920 Hospital of the Joint Logistics Support Force of the Chinese People's Liberation Army (Yunnan)	Yang Liping
The Affiliated Hospital of Guizhou Medical University	Chen Zhangrong
Shengjing Hospital of China Medical University	Sun Zhijun
The University of Hong Kong‐Shenzhen Hospital	Kai‐Hang Yiu
Hunan Provincial People's Hospital	Zheng Zhaofen
First Hospital of Shanxi Medical University	Yang Zhiming
