# Supplementary material for: Nationwide implementation of heart failure therapies: National Heart Failure Center Accreditation Program (HF‐CAP) in China
Source: Eur J Heart Fail. 2025 Sep 22;27(11):1964–76. doi: 10.1002/ejhf.70035 (PMC12765234; doi:10.1002/ejhf.70035)
Supplement: Supplementary file 1 — Appendix S1. Supporting Information. [file EJHF-27-1964-s001.docx]

**Supplementary material**

**Nationwide implementation of heart failure therapies: National Heart Failure Center Accreditation Program (HF-CAP) in China**

Table of Contents

[Supplementary Figure S1 HF-CAP organisational framework 3](#_Toc199333296)

[Supplementary Figure S2 Regional HF care network 4](#_Toc199333297)

[Supplementary Figure S3 HF center accreditation and re-accreditation process 5](#_Toc199333298)

[Supplementary Figure S4 Geographical location, by region, of HF patients included in the present study and hospitalized in centres accredited by HF-CAP 6](#_Toc199333299)

[Supplementary Figure S5 Comparison of prescription rates of different (A) ACEi, (B) ARB and (C) ARNI in patients without contraindications from discharge to 1 year after discharge in HF-CAP centres divided into four groups: before (Pre-accreditation), and 1 (Year 1), 2 (Year 2) or >2 Years (Year 2+) after HF-CAP accreditation of the centre 7](#_Toc199333300)

[Supplementary Figure S6 Proportion of patients receiving different modes of routine follow-up in HF-CAP in the year following discharge, classified by time since accreditation 8](#_Toc199333301)

[Supplementary Figure S7 Association between time since accreditation by HF-CAP and 1-year HF readmission 9](#_Toc199333302)

[Supplemental Figure S8 Association between time since accreditation by HF-CAP and 1-year CV death 10](#_Toc199333303)

[Supplementary Table S1 China HF center accreditation criteria 11](#_Toc199333304)

[Supplementary Table S2 HF-CAP responsibilities and benefits for accredited HF centres 13](#_Toc199333305)

[Supplemental Table S3 Training programme in HF-CAP 15](#_Toc199333306)

[Supplementary Table S4 Patient education program in HF-CAP 16](#_Toc199333307)

[Supplementary Table S5 Data items recorded by each center in the HF-CAP database 18](#_Toc199333308)

[Supplementary Table S6 Characteristics of patients according to accreditation type (tertiary vs. secondary HF centre) 21](#_Toc199333309)

[Supplementary Table S7 Data corresponding to *Figure 2* (comparison of prescription rates of ACEi/ARB/ARNI, beta-blockers, MRA and SGLT2i) 24](#_Toc199333310)

[Supplementary Table S8 Proportion of patients in those without contraindications prescribed each class of GDMT by HF type divided into four groups: before (Pre-accreditation), and 1 (Year 1), 2 (Year 2) or >2 Years (Year 2+) after HF-CAP accreditation of the centre 25](#_Toc199333311)

[Supplementary Table S9 Frequency of different modes of routine follow-up in HF-CAP in the year following discharge, classified by time since accreditation 26](#_Toc199333312)

[Supplementary Table S10 Unadjusted analysis: 1-year readmission among patients hospitalised with HF by time since accreditation 27](#_Toc199333313)

[Supplementary Table S11 Unadjusted 1-Year readmission for worsening HF or CV death according to patient characteristics and divided into four groups: before (Pre-accreditation), and 1 (Year 1), 2 (Year 2) or >2 Years (Year 2+) after HF-CAP accreditation of the centre 28](#_Toc199333314)

[Supplementary Table S12 Sensitivity analysis - OR (95% CI) of the 1-year composite endpoint of readmissions for worsening HF or CV death associated with accreditation status in different models 30](#_Toc199333315)

[Supplementary Table S13 Sensitivity analysis - Odd ratios (95% CI) of the 1-year composite endpoint of readmissions for worsening HF or CV death associated with accreditation status, time-adjusted for PCI, CABG, and hospital level (tertiary/secondary) 32](#_Toc199333316)

[Supplementary Table S14 Sensitivity analysis - OR (95% CI) of the 1-year composite endpoint of readmissions for worsening HF or CV death associated with accreditation status, where patients without echocardiography were excluded 33](#_Toc199333317)

[Supplementary Table S15 Comparison of health status among telephone follow-ups and clinic visits 34](#_Toc199333318)

## Supplementary Figure S1 HF-CAP organisational framework

HF-CAP quality control includes three levels: national, provincial and hospital-level. At the national level, the Board of Directors from the HF-CAP Commission are responsible for: overseeing the implementation of the project and the accreditation and reaccreditation of HF centers; coordinating the relationship between all parties involved in the project; making decisions on project development priorities and resource allocation; resolving major difficulties and internal conflicts in project implementation; and approving project budgets, assessing funding needs and seeking funding opportunities. At the provincial level, Regional HF Center Alliance are responsible for formulating the province’s quality control plan. At the hospital level, a Director appointed by the Board of Directors and Regional HF Center Alliance is responsible for each hospital’s quality control. HF, heart failure; HF-CAP, National Heart Failure Centre Accreditation Program

**
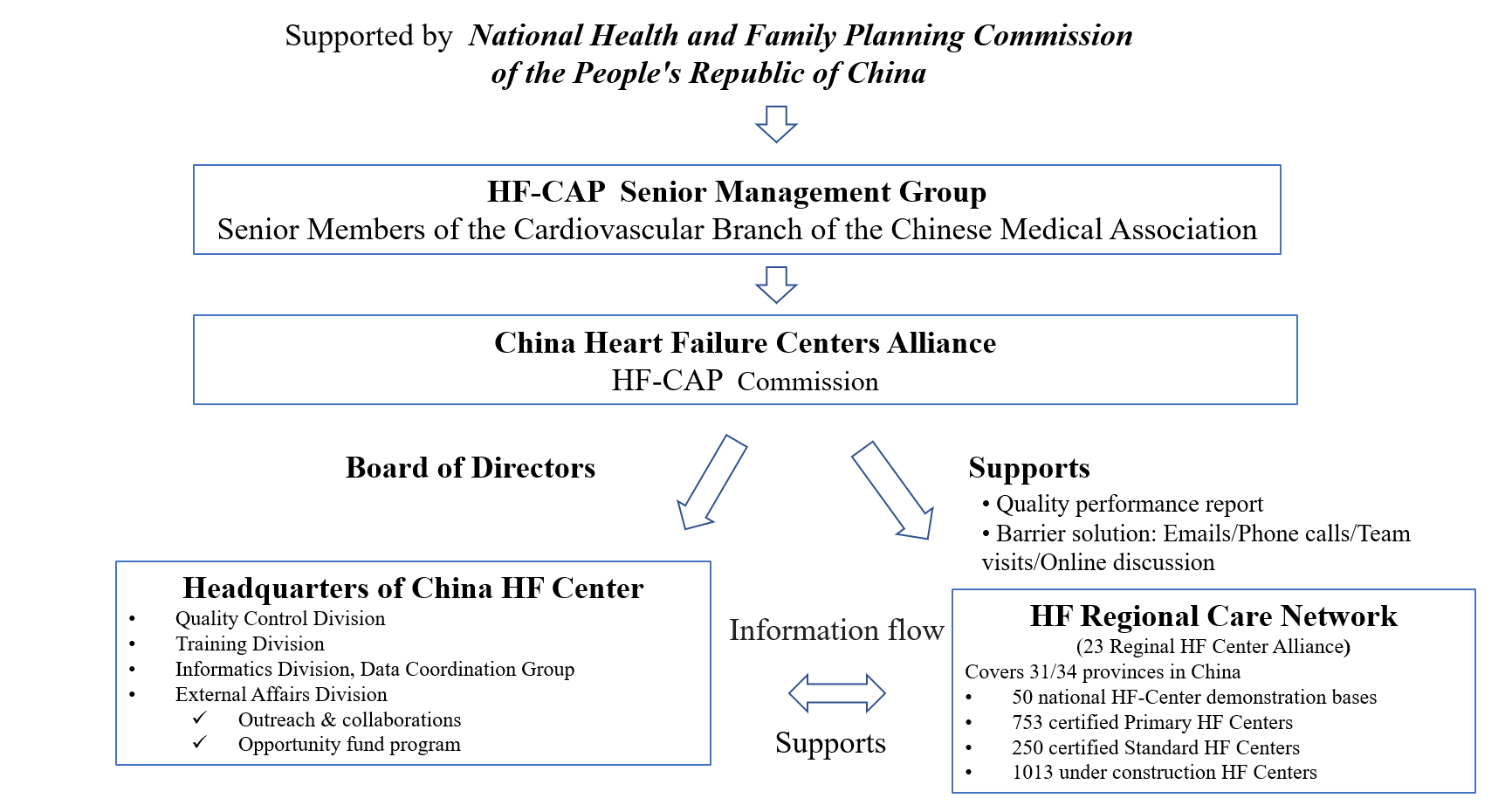
**

## Supplementary Figure S2 Regional HF care network

Regional HF Care Network includes the local hierarchic medical system, which comprises tertiary hospitals (specialized HF centers) and corresponding secondary hospitals (non-specialized HF centers). Regional HF Centers Alliance belongs to the HF-CAP Commission. Hospitals at all levels should ensure continuity of care for patients with HF through 2-way referral. HF, heart failure; HF-CAP, National Heart Failure Centre Accreditation Program

**
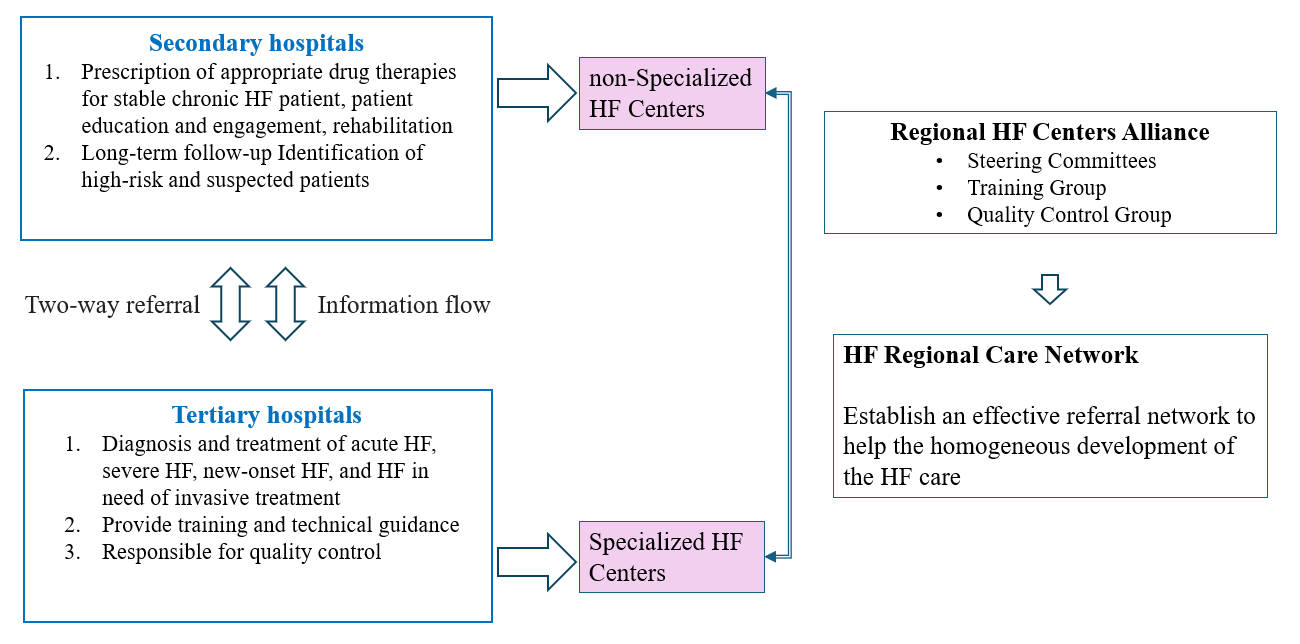
**

## Supplementary Figure S3 HF center accreditation and re-accreditation process

A center is classified as ‘particularly excellent’ if the patient data are very good, e.g. all GDMT ratios are close to 100%; this may due to the centre having a high quality of care, or may be that the data is considered dubious, both cases would result in an on-site visit. HF, heart failure; GDMT, guideline-directed medical therapy

**
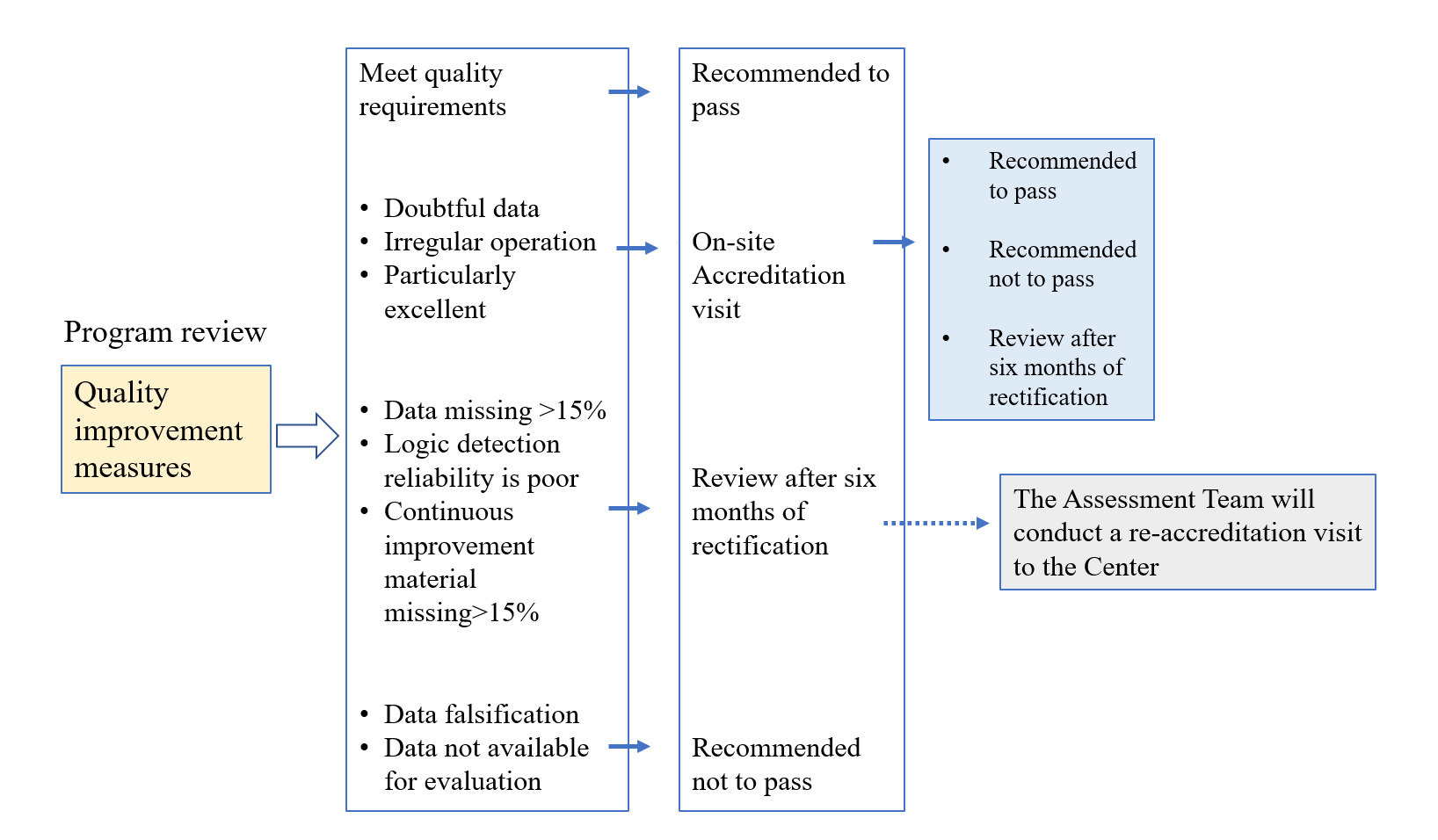
**

## Supplementary Figure S4 Geographical location, by region, of HF patients included in the present study and hospitalized in centres accredited by HF-CAP

HF, heart failure; HF-CAP, National Heart Failure Centre Accreditation Program

**
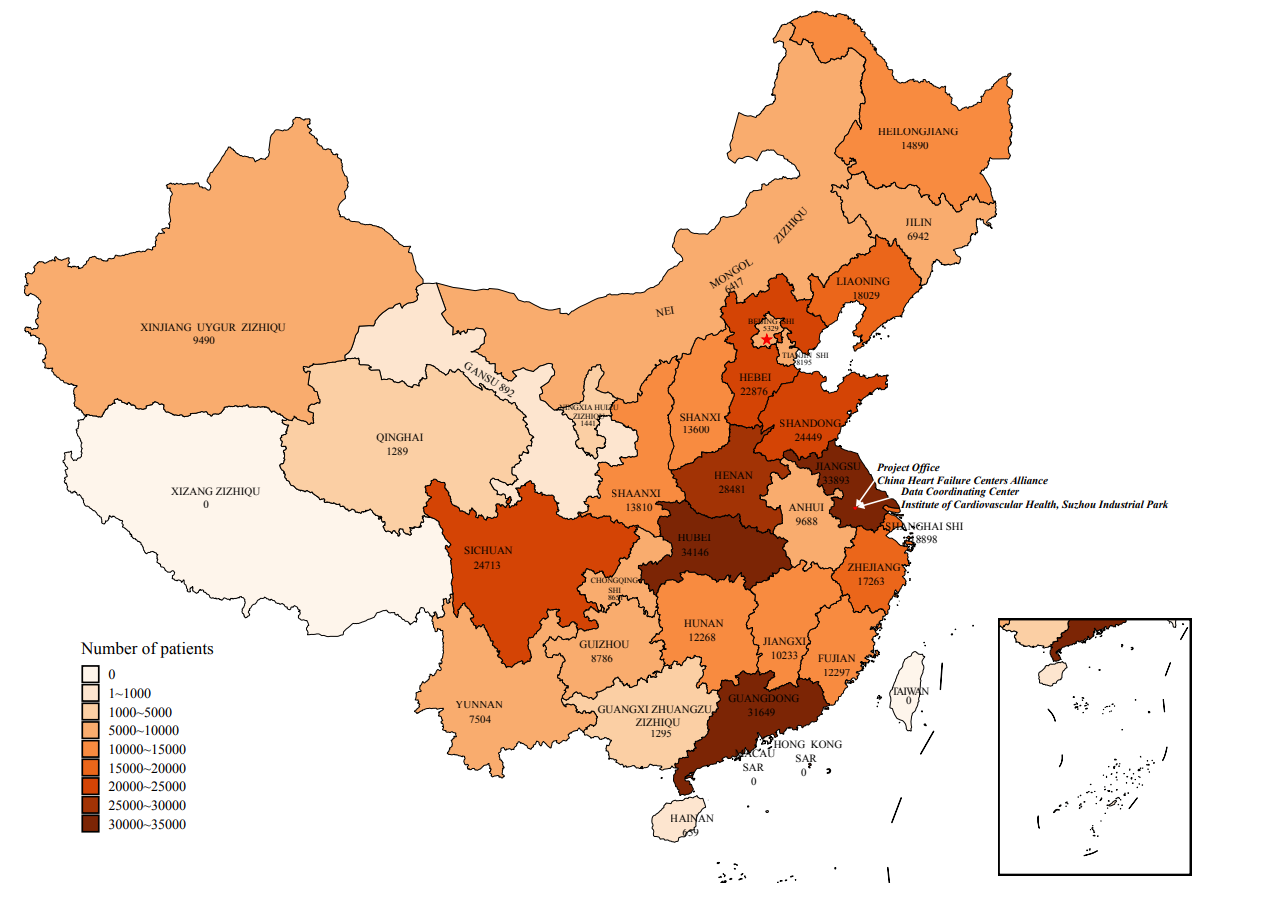
**

Supplementary Figure S5 Comparison of prescription rates of different (A) ACEi, (B) ARB and (C) ARNI in patients without contraindications from discharge to 1 year after discharge in HF-CAP centres divided into four groups: before (Pre-accreditation), and 1 (Year 1), 2 (Year 2) or >2 Years (Year 2+) after HF-CAP accreditation of the centre

ACEi, angiotensin-converting enzyme inhibitors; ARB, angiotensin receptor blockers;
ARNI, angiotensin receptor–neprilysin inhibitors; HF-CAP, National Heart Failure Centre Accreditation Program

## Supplementary Figure S6 Proportion of patients receiving different modes of routine follow-up in HF-CAP in the year following discharge, classified by time since accreditation

HF-CAP, National Heart Failure Centre Accreditation Program


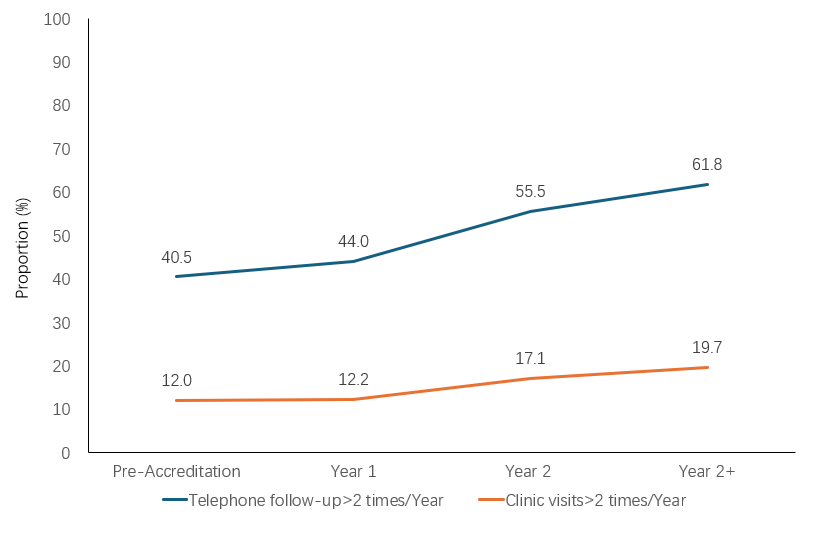


## Supplementary Figure S7 Association between time since accreditation by HF-CAP and 1-year HF readmission

CI, confidence interval; HF, heart failure; HF-CAP, National Heart Failure Centre Accreditation Program; HFmrEF, heart failure with mid-range ejection fraction; HFpEF, heart failure with preserved ejection fraction; HFrEF, heart failure with reduced ejection fraction; Pre–, Pre-accreditation


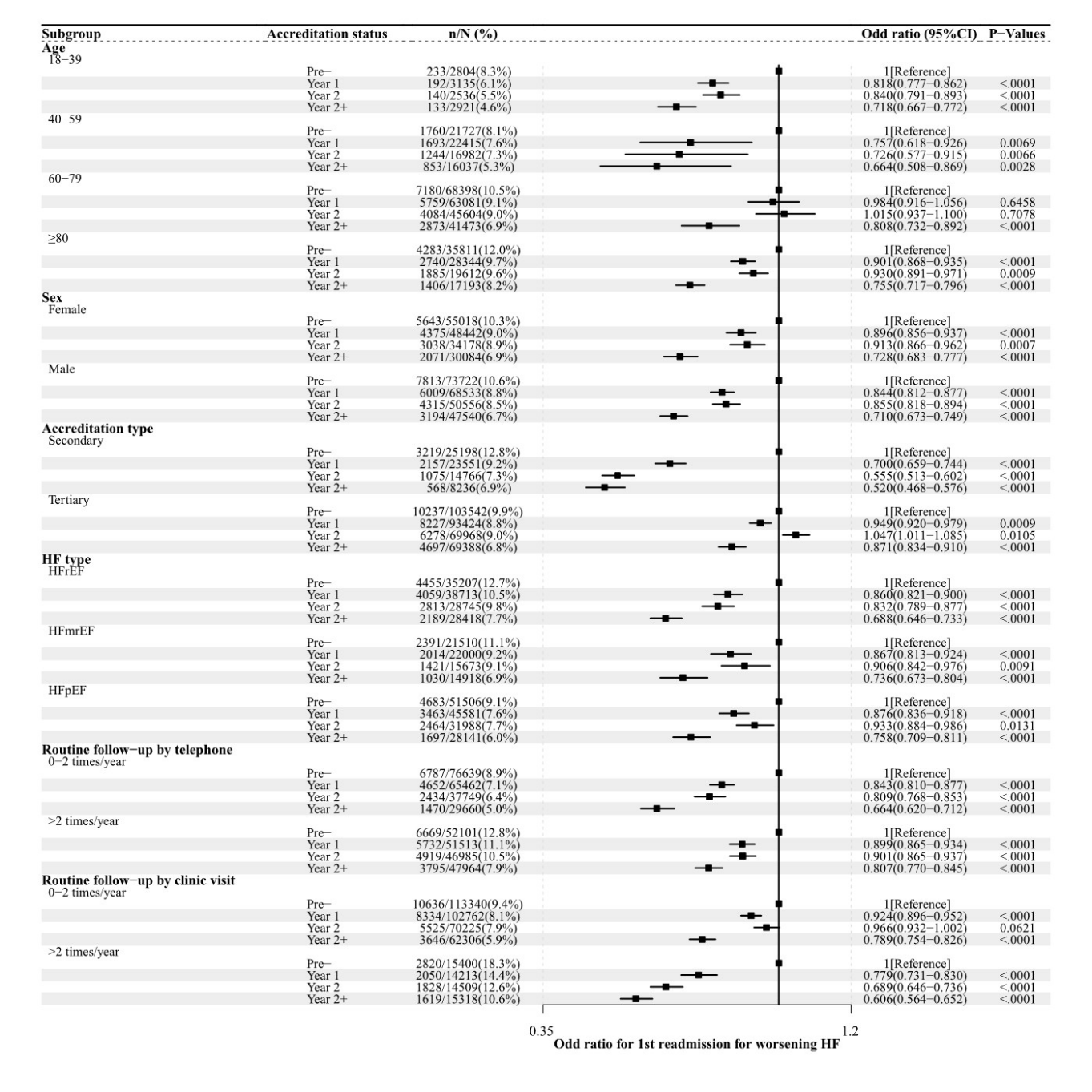


## Supplemental Figure S8 Association between time since accreditation by HF-CAP and 1-year CV death

CV, cardiovascular; HF, heart failure; HF-CAP, National Heart Failure Centre Accreditation Program; HFmrEF, heart failure with mid-range ejection fraction; HFpEF, heart failure with preserved ejection fraction; HFrEF, heart failure with reduced ejection fraction; Pre–, pre-accreditation

**
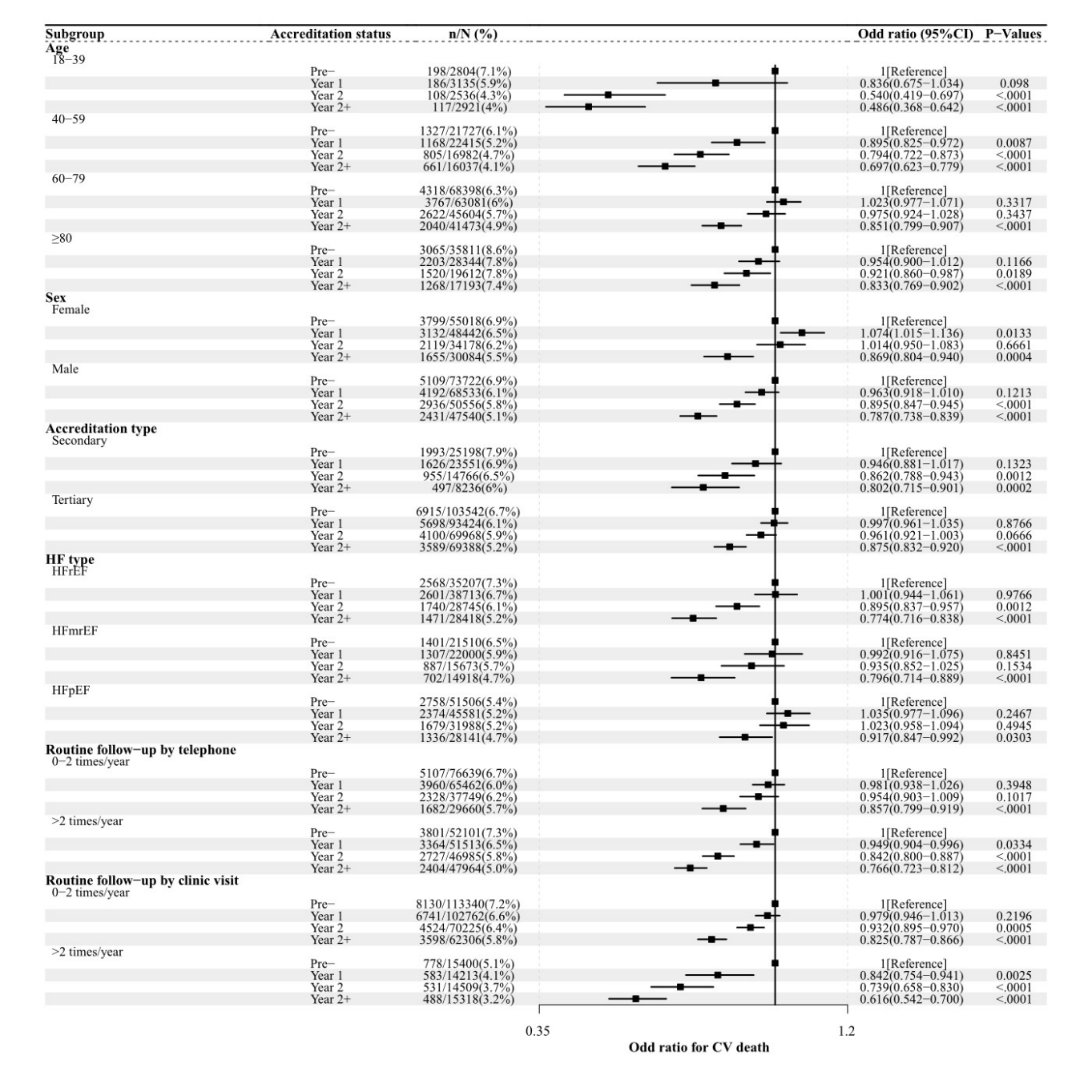
**

## Supplementary Table S1 China HF center accreditation criteria

| **Minimum requirements for accreditation** | **Tertiary hospital** | **Secondary hospital** |
| --- | --- | --- |
| HF hospitalisations/year, *n* | >300 | >100 |
| Diagnosis, % Echocardiography BNP or NT-proBNP | 90 90 | 80 80 |
| Medication at discharge (excluding patients with contraindications) | | |
| HFrEF patients, % ACEi/ARB/ARNi Beta-blockers MRA SGLT2i | 80 80 80 50 | 80 80 80 40 |
| Anticoagulation in patients with atrial fibrillation, % | 60 | 50 |
| Patients with diabetes mellitus using SGLT2i, % | 70 | 70 |
| Follow-up rate, % 1 week  1 month 3 months 1 year | 0 60 50 50 | 80 60 50 50 |
| Medication during follow-up (excluding patients with contraindications), % | | |
| HFrEF patients ACEi/ARB/ARNI Beta-blockers MRA SGLT2i | 80 80 80 60 | 80 80 80 50 |
| Anticoagulation in patients with HF and atrial fibrillation, % | 60 | 50 |
| SGLT2i in patients with HF and diabetes, % | 70 | 70 |
| Drug compliance during follow-up | | |
| HFrEF patients, % ACEi/ARB/ARNi dose compliance rate Beta-blocker dosage compliance rate | 60 60 | 60 60 |
| Percentages reported are the minimum proportion/percentage of patients/events that must be achieved. Accreditation criteria differ based on the hospital type (tertiary or secondary). Accreditation criteria, as quality improvement indictors, consists of five dimensions of qualification based on best practices and health system context: (1) condition of facilities: whether or not they meet the accreditation standards of the National Health Commission of the People's Republic of China for tertiary or secondary hospitals, (2) diagnostic process, (3) hospital care, (4) follow-up rate, and (5) post-discharge care  ACEi, angiotensin-converting enzyme inhibitor; ARB, angiotensin receptor blocker; ARNI, angiotensin receptor–neprilysin inhibitor; BNP, brain natriuretic peptide; HF, heart failure; HFrEF, heart failure with reduced ejection fraction; MRA, mineralocorticoid receptor antagonist; NT-proBNP, *N*-terminal pro-B-type natriuretic peptide; SGLT2i, sodium–glucose cotransporter 2 inhibitor | | |

## Supplementary Table S2 HF-CAP responsibilities and benefits for accredited HF centres

| ***Responsibilities***   1. Achieve key indicators of HF management performance; continuous improvement of the quality of HF care. 2. Develop and implement a strategy for data collection; ensure timely and accurate data collection; maintain project-related data and materials. 3. Actively participate in collaborative training projects and regional forums to discuss ways to practice quality of care improvement. 4. Tertiary centres are obliged to mentor secondary centres and provide on-site learning and training.   ***Benefits***  HF centers can take advantage of the programme’s multiple resources and improve HF quality of care.   1. Accreditation is a measure of HF care quality. Only HF centres that maintain high standards for performance outcomes can earn and maintain accreditation. As a result, accreditation signals to prospective patients admitted to those centres that they meet national standards. 2. Accreditation and National/Regional care network aid: technical support, barrier solutions and 2-way referrals. In addition, many HF clinical research programmes require investigators to be part of an accredited institution. 3. Training and educational resources: access to a variety of online educational resources offered by the programme, including web-based training courses, learning materials, the latest national and international guidelines and explanations of those guidelines by HF-CAP experts and discussions of typical cases. HF centre members have the opportunity to receive funding to attend regional programme training sessions and exchange workshops to learn about the programme’s progress and work plans, share experiences in quality of care improvement and discuss best practices together. 4. On-site exchanges: as needed, centres of excellence offer short visits for familiarity with optimal quality of care and to promote the dissemination of experience.   Concerning the HF-CAP database, the project team organize information sessions attended by each HF centre to explain how to record the centre’s data into the HF-CAP database; centre data can eventually be directly analyzed by each centre for their research and management analysis. |
| --- |
| HF, heart failure; HF-CAP, National Heart Failure Center Accreditation Program |

## Supplemental Table S3 Training programme in HF-CAP

| ***Training for multidisciplinary team at tertiary HF Centres***   1. Experts of **China HF Alliance and Regional HF Alliance** help centres to identify the main problems of medical quality, proposing improvement measures and promoting problem-oriented improvement of medical quality. 2. Regional training courses: Including summary of project progress, training and experience exchange. 3. On-site communication: According to needs, the medical staff of the HF centre will be invited to the centres with the highest quality of care (= center of excellence) for on-site visits and exchanges to gain expertise. 4. HF-CAP web-based courses (documents, slides, videos, etc.): Participating centres can browse and download through the project website with the project’s proprietary user name and password. 5. Other educational materials: Academic conference presentations and exchanges, brochures, and pocket guides with the program logo, etc. |
| --- |
| HF, heart failure; HF-CAP, National Heart Failure Center Accreditation Program |

## Supplementary Table S4 Patient education program in HF-CAP

| **Intervention** | **Details** |
| --- | --- |
| Exercise rehabilitation guidance | - Recommend exercise of different intensities according to the patient’s cardiac function - Reduce sedentary behaviour, increase moderate intensity exercise and pay attention to gradual progress - Encourage patients to actively participate in various recommended forms of exercise and community group activities - Advise on how to avoid or reduce injuries during exercise |
| Sodium restriction | - In acute HF with volume overload, restrict sodium intake to <2 g/d - Strict sodium restriction is not recommended in patients with mild or stable HF |
| Water restriction | - Patients with severe HF: 1.5 to 2.0 L/d - Patients with mild to moderate symptomatic HF do not benefit from routine fluid restriction |
| Nutrition and diet | - Low fat - Smoking and alcohol cessation - Weight loss in obese patients - Nutritional support in patients with cardiac cachexia |
| Weight monitoring | - Sudden weight gain of more than 2 kg in 3 days should be considered  as water and sodium retention |
| Blood pressure, lipid, and blood glucose control | - Control blood pressure, blood lipids and blood glucose within  appropriate range |
| Psychologic and spiritual guidance | - Advise to maintain a positive and optimistic attitude - Comprehensive emotional interventions such as psychological  de-escalation - Consider anti-anxiety medications or antidepressants if necessary  and appropriate |
| Guidance on medication use | - Explain in detail the medication list, including the name, dose, time, frequency, purpose of medication, adverse reactions, precautions, etc. - Print out the medication list for patients to improve their compliance  with medication |
| Follow-up arrangements | - Detailed explanation of the schedule and purpose of follow-up visits - Suggest that follow-up visits can be personalized, if needed, such as 2 weeks, 1 month, 3 months, 6 months, 1 year or 2 years after discharge - Timely medical intervention according to the results of follow-up visits |
| Self-assessment and management of symptoms | - How to detect the deterioration of HF symptoms early and how to deal with it |
| Graphs, pictures, and videos are produced to provide understandable and accessible information to patients with HF and their families.  HF, heart failure; HF-CAP, National Heart Failure Center Accreditation Program | |

## Supplementary Table S5 Data items recorded by each center in the HF-CAP database

| **Category** | **Example elements** |
| --- | --- |
| During hospitalisation | |
| Medical care in facility | Admission date, discharge date |
| Demographics | Age, sex, ethnicity, occupation, education level, marital status, type of medical insurance |
| Medical history | Alcohol consumption, smoking, history of HF, family CVD history |
| Risk factors | Poor treatment compliance, arrhythmia, blood loss or anemia, infection, coronary artery disease, excessive water intake, fatigue, stress, pregnancy, hypertension, abnormal renal function, pulmonary embolism, hyperthyroidism |
| Comorbidities/conditions | Hypertension, coronary heart disease (myocardial infarction), PCI/CABG, atrial fibrillation, stroke/TIA, peripheral arterial disease, diabetes, dyslipidemia, asthma/COPD, chronic kidney disease, thyroid dysfunction, malignancy, depression/anxiety |
| Clinical symptoms | Dyspnea, oedema, fatigue, mobility, self-care, pain |
| Physical examination | Weight, height, blood pressure, heart rate, SpO2, pulmonary rales, elevated jugular venous pressure, peripheral oedema, etc. |
| Laboratory test | BNP/NT-proBNP, Na+, K+, creatinine, blood lipid, uric acid, haemoglobin, thyroid function, fasting blood glucose, HbA1c |
| ECG/echocardiography | ECG: heart rate and rhythm, LBBB, QRS duration Echocardiography: LVEF, left ventricular end-diastolic diameter, cardiac function, NYHA class, 6-minute walk test |
| Medications for HF | ACEis, ARBs, ARNIs, beta-blockers, aldosterone receptor antagonists, ivabradine, diuretics, digoxin, statins, antiplatelet agents, anticoagulants, nitrates, calcium channel blockers, antiarrhythmic drugs (amiodarone, etc.), intravenous inotropic drugs, Qili Qiangxin capsules |
| Other drugs | Non-steroidal anti-inflammatory drugs, antidiabetic drugs, antidepressants, thyroid hormone replacement therapy, etc. |
| Implanted device | ICD, CRT, pacemaker, etc. |
| Discharge | Date of discharge, principal discharge diagnosis, diagnosis code,  procedure code |
| In-hospital clinical events | Death and cause of death, cardiogenic shock, cardiac arrest, stroke |
| After discharge | |
| Follow-up visit | Symptoms, signs, laboratory tests, electrocardiogram, echocardiography, medication regimen adjustment |
| New events | Death and cause of death, hospitalisation and cause of hospitalisation,  new implanted device, etc. |
| Feedback | |
| Data clarifications | Collects and directs all additional comments, questions, and further clarifications regarding the data |
| Data accuracy and completeness are ensured by the following four strategies. (1) HF centres should have full-time or part-time data managers who are responsible for the management of patients’ medical files, including data entry, backup, update, sorting and statistics. (2) The data managers must pass the training course and obtain the certification; in addition, a free 24 h hotline service by DCG is provided to ensure that data-entry questions are resolved in a timely manner. Data must be entered within 30 days of discharge. (3) A standardized web-based data collection platform will perform approximately 80% of the error checking during the first entry of the data. Error checks identify missing data, missing forms, out-of-range values and illogical or inconsistent data. Additional error checks will be executed on a nightly basis (or on demand) to identify more complex errors such as illogical or inconsistent data across visits. The DCG data specialist will query the site data managers using an error tracking application and the data managers will edit the database appropriately. If no feedback is obtained within 1 week after the report is issued, DCG will arrange additional on-site guidance and assessment. (4) The data analysis team in DCG help each centre with the data analysis on a regular basis.  ACEi, angiotensin-converting enzyme inhibitor; ARB, angiotensin receptor blocker; ARNI, angiotensin receptor–neprilysin inhibitor; BNP, brain natriuretic peptide; CABG, coronary artery bypass graft; COPD, chronic obstructive pulmonary disease; CRT, cardiac resynchronization therapy; CVD, cardiovascular disease; DCG, Data Coordination Group; ECG, electrocardiogram; HbA1c, haemoglobin A1C; HF, heart failure; HF-CAP, National Heart Failure Center Accreditation Program; ICD, implantable cardioverter defibrillator; K+, potassium; LBBB, left bundle branch block; LVEF, left ventricular ejection fraction; Na+, sodium; NT-proBNP, *N*-terminal pro-B-type natriuretic peptide; NYHA, New York Heart Association; PCI, percutaneous coronary intervention; QRS, Q wave, R wave and S wave; SpO2, saturation of peripheral oxygen; TIA, transient ischemic attack | |

## Supplementary Table S6 Characteristics of patients according to accreditation type (tertiary vs. secondary HF centre)

|  | **Tertiary hospital (*n* = 336 322)** | **Secondary hospital (*n* = 71 751)** |
| --- | --- | --- |
| Demographic Female, *n* (%) Age (y), mean±SD  Median (IQR) | 133 817 (39.8) 69.0±13.4 70 (61, 79) | 33 905 (47.3) 72.3±12.3 74 (65, 81) |
| History, comorbidities, *n* (%) Hypertension  Type 2 diabetes mellitus  Hyperlipidemia Obesity Ischaemic heart disease  Prior myocardial infarction  Atrial fibrillation Stroke Renal insufficiency COPD or asthma Depression/Anxiety Anaemia | 195 028 (58.0) 95 080 (28.3) 49 656 (14.8) 63 274 (18.8) 203 925 (60.6) 81 921 (24.4) 109 353 (32.5) 40 370 (12.0) 43 078 (12.8) 29 870 (8.9) 4208 (1.3) 72 422 (21.5) | 42 242 (58.9) 16 502 (23.0) 9158 (12.8) 12 003 (16.7) 46 579 (64.9) 11 857 (16.5) 25 362 (35.4) 9477 (13.2) 7964 (11.1) 9207 (12.8) 1357 (1.9) 14 011 (19.5) |
| History, device/procedure, *n* (%) PCI CABG Pacemaker ICD CRT-P CRT-D | 69 707 (20.7) 2873 (.9) 12 759 (3.8) 3298 (1.0) 1137 (.3) 3014 (.9) | 14 960 (20.9) 287 (.4) 1392 (1.9) 141 (.2) 69 (.1) 130 (.2) |
| Vitals on admission, mean±SD BMI (kg/m^2^) SBP (mmHg) DBP (mmHg) Heart rate (bpm) | 23.7±3.6 130.7±24.1 77.3±14.1 83.0±20.4 | 23.2±3.6 136.1±24.9 79.7±14.2 84.5±21.5 |
| Laboratory on admission NT-proBNP (pg/mL)  Patients assessed, *n* (%)^a^  Value (pg/mL), median (IQR) LVEF  Patients, *n* (%)  Value (%), mean±SD | 268 029 (79.7) 2273.6 (918, 5564)  301 275 (89.6) 46.1±13.7 | 49 375 (68.8) 2128.6 (826, 5363)  61 125 (85.2) 49.2±13.3 |
| NYHA class (closest to admission), *n* (%) I II III IV Missing | 4940 (1.5) 35 021 (10.4) 82 857 (24.6) 55 724 (16.6) 157 780 (46.9) | 766 (1.1) 8161 (11.4) 28 072 (39.1) 20 526 (28.6) 14 226 (19.8) |
| HF type, *n* (%) HFrEF HFmrEF HFpEF HF unclassifiable^b^ | 113 641 (33.8) 61 025 (18.1) 126 609 (37.7) 35 047 (10.4) | 17 442 (24.3) 13 076 (18.2) 30 607 (42.7) 10 626 (14.8) |
| Admission year, *n* (%) 2017 2018 2019 2020 2021 2022 | 12 830 (3.8) 51 543 (15.3) 81 533 (24.2) 73 794 (21.9) 63 442 (18.9) 53 180 (15.8) | 315 (.4) 2193 (3.1) 9925 (13.8) 16 841 (23.5) 21 690 (30.2) 20 787 (29.0) |
| ^a^Number of patients (%) with NT-proBNP concentrations at index hospitalization. ^b^Missing baseline LVEF  BMI, body mass index; bpm, beats per minute; CABG, coronary artery bypass graft; COPD, chronic obstructive pulmonary disease; CRT-D, cardiac resynchronization therapy defibrillator; CRT-P, cardiac resynchronization therapy pacemaker; DBP, diastolic blood pressure; HF, heart failure; HFmrEF, heart failure with mid-range ejection fraction; HFpEF, heart failure with preserved ejection fraction; HFrEF, heart failure with reduced ejection fraction; ICD, implantable cardioverter defibrillator; IQR, interquartile range; LVEF, left ventricular ejection fraction; NT-proBNP, *N*-terminal pro-B-type natriuretic peptide; NYHA, New York Heart Association; PCI, percutaneous coronary intervention; SBP, systolic blood pressure; SD, standard deviation | | |

## Supplementary Table S7 Data corresponding to *Figure 2* (comparison of prescription rates of ACEi/ARB/ARNI, beta-blockers, MRA and SGLT2i)

|  | **Total** | **Pre-accreditation** | **Post-accreditation** | | |
| --- | --- | --- | --- | --- | --- |
|  |  |  | **Year 1** | **Year 2** | **Year 2+** |
| At discharge ACEi/ARB/ARNI  Beta-blockers MRA SGLT2i | 88.4 91.5 89.8 4.4 | 86.3 90.9 91.8 .1 | 88.6 91.2 90.1 2.4 | 87.4 90.7 86.2 5.3 | 92.5 93.7 90.1 15.5 |
| 30 days after discharge ACEi/ARB/ARNI  Beta-blockers MRA SGLT2i | 75.4 77.3 72.6 6.2 | 69.9 71.7 70.2 1.2 | 74.6 76.1 70.4 4.0 | 77.3 80.0 73.1 6.9 | 83.8 85.2 80.0 18.5 |
| 90 days after discharge ACEi/ARB/ARNI  Beta-blockers MRA SGLT2i | 70.8 72.8 67.2 6.2 | 65.3 67.1 64.5 1.4 | 69.2 70.6 64.1 4.3 | 73.6 76.4 68.6 6.4 | 79.6 81.3 75.0 18.8 |
| 1 year after discharge ACEi/ARB/ARNI  Beta-blockers MRA SGLT2i | 60.2 62.1 55.0 5.6 | 52.0 53.6 49.2 2.7 | 58.8 60.3 52.8 4.5 | 66.2 68.5 59.7 5.6 | 72.2 74.4 66.7 15.5 |
| ACEi, angiotensin-converting enzyme inhibitor; ARB, angiotensin receptor blocker; ARNI, angiotensin receptor–neprilysin inhibitor; MRA, mineralocorticoid receptor antagonist; SGLT2i, sodium–glucose cotransporter 2 inhibitor | | | | | |

## Supplementary Table S8 Proportion of patients in those without contraindications prescribed each class of GDMT by HF type divided into four groups: before (Pre-accreditation), and 1 (Year 1), 2 (Year 2) or >2 Years (Year 2+) after HF-CAP accreditation of the centre

|  | **Total** | **All patients** | | | |  | **HFrEF** | | | |  | **HFmrEF** | | | |  | **HFpEF** | | | |
| --- | --- | --- | --- | --- | --- | --- | --- | --- | --- | --- | --- | --- | --- | --- | --- | --- | --- | --- | --- | --- |
|  |  | **Pre-accreditation** | **Post-accreditation** | | |  | **Pre-accreditation** | **Post-accreditation** | | |  | **Pre-accreditation** | **Post-accreditation** | | |  | **Pre-accreditation** | **Post-accreditation** | | |
|  |  |  | **Y1** | **Y2** | **Y2+** |  |  | **Y1** | **Y2** | **Y2+** |  |  | **Y1** | **Y2** | **Y2+** |  |  | **Y1** | **Y2** | **Y2+** |
| At discharge, % ACEi/ARB/ARNI  Beta-blockers MRA SGLT2i | 88.4 91.5 89.8 4.4 | 86.3 90.9 91.8 .1 | 88.6 91.2 90.1 2.4 | 87.4 90.7 86.2 5.3 | 92.5 93.7 90.1 15.5 |  | 89.8 94.1 96.2 .2 | 92.3 94.4 95.3 3.0 | 92.4 94.8 92.8 6.5 | 96.5 96.8 95.6 19.7 |  | 89.3 93.2 94.5 .1 | 90.9 93.2 91.6 3.0 | 89.3 92.0 86.5 6.2 | 94.0 94.7 90.6 16.4 |  | 85.8 90.5 89.9 .1 | 86.9 89.3 85.92 .0 | 84.0 88.0 80.6 4.3 | 89.0 91.3 84.5 12.0 |
| 30 days after discharge, % ACEi/ARB/ARNI  Beta-blockers MRA SGLT2i | 75.4 77.3 72.6 6.2 | 69.9 71.7 70.2 1.2 | 74.6 76.1 70.4 4.0 | 77.3 80.0 73.1 6.9 | 83.8 85.2 80.0 18.5 |  | 73.2 74.7 76.5 1.6 | 77.8 78.6 77.0 5.1 | 81.6 83.4 80.0 7.8 | 87.0 88.0 85.7 23.1 |  | 73.9 76.1 75.1 1.3 | 76.9 78.6 72.1 4.7 | 78.4 81.4 73.4 7.8 | 85.8 86.8 80.6 18.9 |  | 68.4 70.2 65.4 .9 | 72.1 74.3 64.8 3.1 | 73.6 77.2 66.5 5.7 | 80.6 82.4 74.0 14.9 |
| 90 days after discharge, % ACEi/ARB/ARNI  Beta-blockers MRA SGLT2i | 70.8 72.8 67.2 6.2 | 65.3 67.1 64.5 1.4 | 69.2 70.6 64.1 4.3 | 73.6 76.4 68.6 6.4 | 79.6 81.3 75.0 18.8 |  | 68.1 69.5 70.5 1.6 | 71.9 72.7 70.0 5.5 | 78.2 80.1 75.7 7.2 | 82.5 83.8 80.5 23.5 |  | 69.2 71.3 69.1 1.5 | 71.3 72.9 65.2 4.9 | 75.5 78.2 69.1 7.1 | 81.5 82.8 75.1 18.9 |  | 63.8 65.7 59.9 1.1 | 67.0 69.0 59.2 3.3 | 69.4 73.1 61.9 5.3 | 76.4 78.7 69.0 14.9 |
| 1 year after discharge, % ACEi/ARB/ARNI  Beta-blockers MRA SGLT2i | 60.2 62.1 55.0 5.6 | 52.0 53.6 49.2 2.7 | 58.8 60.3 52.8 4.5 | 66.2 68.5 59.7 5.6 | 72.2 74.4 66.7 15.5 |  | 53.7 55.2 53.6 2.9 | 61.7 62.4 57.9 5.6 | 69.5 71.0 65.3 6.4 | 74.5 76.2 71.4 20.7 |  | 54.1 56.6 51.9 2.8 | 60.0 61.8 53.2 5.1 | 68.0 70.2 59.7 6.1 | 75.1 76.9 67.6 14.3 |  | 51.6 53.4 45.8 2.1 | 56.8 59.0 48.7 3.3 | 62.6 65.8 53.9 4.2 | 68.9 72.0 60.9 11.2 |
| ACEi, angiotensin-converting enzyme inhibitor; ARB, angiotensin receptor blocker; ARNI, angiotensin receptor–neprilysin inhibitor; GDMT, guideline-directed medical therapy; HF, heart failure; HF-CAP, National Heart Failure Center Accreditation Program; HFmrEF, heart failure with mid-range ejection fraction; HFpEF, heart failure with preserved ejection fraction; HFrEF, heart failure with reduced ejection fraction; MRA, mineralocorticoid receptor antagonist; SGLT2i, sodium–glucose cotransporter 2 inhibitor; Y, year | | | | | | | | | | | | | | | | | | | | |

## Supplementary Table S9 Frequency of different modes of routine follow-up in HF-CAP in the year following discharge, classified by time since accreditation

| **Routine follow-up** | **All patients (*n* = 408 073)** | **Pre-accreditation (*n* = 128 740)** | **Year 1 (*n* = 116 975)** | **Year 2 (*n* = 84 734)** | **Year 2+ (*n* = 77 624)** |
| --- | --- | --- | --- | --- | --- |
| Telephone follow-up, *n* (%) 0–2 visits >2 visits | 209 510 (51.3) 198 563 (48.7) | 76 639 (59.5) 52 101 (40.5) | 65 462 (56.0) 51 513 (44.0) | 37 749 (44.6) 46 985 (55.5) | 29 660 (38.2) 47 964 (61.8) |
| Clinic visits, *n* (%) 0–2 visits >2 visits | 348 633 (85.4) 59 440 (14.6) | 113 340 (88.0) 15 400 (12.0) | 102 762 (87.9) 14 213 (12.2) | 70 225 (82.9) 14 509 (17.1) | 62 306 (80.3) 15 318 (19.7) |
| *P* for trends < .001  HF-CAP, National Heart Failure Center Accreditation Program | | | | | |

## Supplementary Table S10 Unadjusted analysis: 1-year readmission among patients hospitalised with HF by time since accreditation

| **Outcome** | **Pre-accreditation (*n* = 128 740)** | **Post-accreditation** | | | ***P* for trend^a^** |
| --- | --- | --- | --- | --- | --- |
|  |  | **Year 1 (*n* = 116 975)** | **Year 2 (*n* = 84 734)** | **Year 2+ (*n* = 77 624)** |  |
| 1st readmission for worsening HF or/and CV death Patients, *n* % (95% CI) | 19 380 15.1 (14.9–15.3) | 15 309 13.1 (12.9–13.3) | 10 632 12.5 (12.3–12.8) | 8286 10.7 (10.5–10.9) | < .001 |
| 1st readmission for worsening HF Patients, *n* % (95% CI) | 13 456 10.5 (10.3–10.6) | 10 384 8.9 (8.7–9.0) | 7353 8.7 (8.5–8.9) | 5265 6.8 (6.6–7.0) | < .001 |
| CV death No. of patients % (95% CI) | 8908 6.9 (6.8–7.1) | 7324 6.3 (6.1–6.4) | 5055 6.0 (5.8–6.1) | 4086 5.3 (5.1–5.4) | < .001 |
| Time since accreditation groups: Pre-accreditation: 6–12 months before accreditation; Post-accreditation Year 1: >0 and ≤12 months after accreditation; Post-accreditation Year 2: >12 and ≤24 months after accreditation; Post-accreditation Year 2+: >24 months after accreditation. ^a^Calculated using Cochran–Armitage Trend test  CI, confidence interval; CV, cardiovascular; HF, heart failure | | | | | |

## Supplementary Table S11 Unadjusted 1-Year readmission for worsening HF or CV death according to patient characteristics and divided into four groups: before (Pre-accreditation), and 1 (Year 1), 2 (Year 2) or >2 Years (Year 2+) after HF-CAP accreditation of the centre

|  | **Pre-accreditation, *n* (% [95% CI])** | **Post-accreditation, *n* (% [95% CI])** | | | ***P*** **for trend^a^** |
| --- | --- | --- | --- | --- | --- |
|  |  | **Year 1, *n* (% [95% CI])** | **Year 2** | **Year 2+** |  |
| All patients Patients, *n* 1st readmission for worsening HF or/and CV death 1st readmission for worsening HF CV death | 128 740 19 380 (15.1 [14.9–15.3])  13 456 (10.5 [10.3–10.6]) 8908 (6.9 [6.8–7.1]) | 116 975 15 309 (13.1 [12.9–13.3])  10 384 (8.9 [8.7–9.0]) 7324 (6.3 [6.1–6.4]) | 84 734 10 632 (12.5 [12.3–12.8])  7353 (8.7 [8.5–8.9]) 5055 (6.0 [5.8–6.1)] | 77 624 8286 (10.7 [10.5–10.9])  5265 (6.8 [6.6–7.0]) 4086 (5.3 (5.1–5.4]) | < .001  < .001 < .001 |
| Sex *Female* Patients, *n* 1st readmission for worsening HF or/and CV death 1st readmission for worsening HF CV death *Male* Patients, *n* 1st readmission for worsening HF or/and CV death 1st readmission for worsening HF CV death | 55 018 8181 (14.9 [14.6–15.2])  5643 (10.3 [10.0–10.5]) 3799 (6.9 [6.7–7.1])  73 722 11 199 (15.2 [14.9–15.5])  7813 (10.6 [10.4–10.8]) 5109 (6.9 (6.8–7.1]) | 48 442 6481 (13.4 [13.1–13.7])  4375 (9.0 [8.8–9.3]) 3132 (6.5 [6.3–6.7])  68 533 8828 (12.9 [12.6–13.1])  6009 (8.8 [8.6–9.0]) 4192 (6.1 [5.9–6.3]) | 34 178 4392 (12.9 [12.5–13.2])  3038 (8.9 [8.6–9.2]) 2119 (6.2 [6.0–6.5])  50 556 6240 (12.3 [12.1–12.6])  4315 (8.5 [8.3–8.8]) 2936 (5.8 [5.6–6.0]) | 30 084 3292 (10.9 [10.6–11.3])  2071 (6.9 [6.6–7.2]) 1655 (5.5 [5.3–5.8])  47 540 4994 (10.5 [10.2–10.8])  3194 (6.7 [6.5–7.0]) 2431 (5.1 [4.9–5.3]) | < .001  < .001 < .001   < .001  < .001 < .001 |
| HF type *HFrEF* Patients, *n* 1st readmission for worsening HF or/and CV death 1st readmission for worsening HF CV death *HFmrEF* Patients, *n* 1st readmission for worsening HF or/and CV death 1st readmission for worsening HF CV death *HFpEF* Patients, *n* 1st readmission for worsening HF or/and CV death 1st readmission for worsening HF CV death | 35 207 6019 (17.1 [16.7–17.5])  4455 (12.7 [12.3–13]) 2568 (7.3 [7.0–7.6])  21 510 3255 (15.1 [14.7–15.6])  2391 (11.1 [10.7–11.5]) 1401 (6.5 [6.2–6.9])  51 506 6540 (12.7 [12.4–13.0])  4683 (9.1 [8.9–9.3]) 2758 (5.4 [5.2–5.6]) | 38 713 5681 (14.7 [14.32–15.00])  4059 (10.5 [10.20–10.80]) 2601 (6.7 [6.50–7.00])  22 000 2868 (13.0 [12.60–13.50])  2014 (9.2 [8.80–9.50]) 1307 (5.9 [5.60–6.30])  45 581 5099 (11.2 [10.90–11.50])  3463 (7.6 [7.40–7.80]) 2374 (5.2 [5.00–5.40]) | 28 745 3858 (13.4 [13.03–13.80])  2813 (9.8 [9.40–10.10]) 1740 (6.1 [5.80–6.30])  15 673 1987 (12.7 [12.20–13.20])  1421 (9.1 [8.60–9.50]) 887 (5.7 [5.30–6.00])  31 988 3569 (11.2 [10.80–11.50])  2464 (7.7 [7.40–8.00]) 1679 (5.3 [5.00–5.50]) | 28 418 3222 (11.3 [10.97–11.70])  2189 (7.7 [7.40–8.00]) 1471 (5.2 [4.90–5.40])  14 918 1541 (10.3 [9.90–10.80])  1030 (6.9 [6.50–7.30]) 702 (4.7 [4.40–5.10)])  28 141 2703 (9.6 [9.30–10.00])  1697 (6.0 [5.80–6.30]) 1336 (4.8 [4.50–5.00]) | < .001  < .001 < .001   < .001  < .001 < .001   < .001  < .001 < .001 |
| Hospital expertise in HF *Secondary* Patients, *n* 1st readmission for worsening HF or/and CV death 1st readmission for worsening HF CV death *Tertiary* Patients, *n* 1st readmission for worsening HF or/and CV death 1st readmission for worsening HF CV death | 25 198 4529 (18.0 [17.5–18.5])  3219 (12.8 [12.4–13.2]) 1993 (7.9 [7.6–8.3])  103 542 14 851 (14.3 [14.1–14.6])  10 237 (9.9 [9.7–10.1]) 6915 (6.7 [6.5–6.8]) | 23 551 3411 (14.5 [14.0–14.9])  2157 (9.2 [8.8–9.5]) 1626 (6.9 [6.6–7.2])  93 424 11 898 (12.7 [12.5–13.0])  8227 (8.8 [8.6–9.0]) 5698 (6.1 [6.0–6.3]) | 14 766 1853 (12.5 [12.0–13.1])  1075 (7.3 [6.9–7.7]) 955 (6.5 [6.1–6.9])  69 968 8779 (12.5 [12.3–12.8])  6278 (9.0 [8.8–9.2]) 4100 (5.9 [5.7–6.0]) | 8236 975 (11.8 [11.2–12.6])  568 (6.9 [6.4–7.5]) 497 (6.0 [5.5–6.6])  69 388 7311 (10.5 [10.3–10.8])  4697 (6.8 [6.6–7.0]) 3589 (5.2 [5.0–5.3]) | < .001  < .001 < .001   < .001  < .001 < .001 |
| Data presented as *n* (% [95% CI]). ^a^Calculated using Cochran–Armitage Trend tests  CI, confidence interval; CV, cardiovascular; HF, heart failure; HF-CAP, National Heart Failure Center Accreditation Program; HFmrEF, heart failure with mid-range ejection fraction; HFpEF, heart failure with preserved ejection fraction; HFrEF, heart failure with reduced ejection fraction | | | | | |

## Supplementary Table S12 Sensitivity analysis - OR (95% CI) of the 1-year composite endpoint of readmissions for worsening HF or CV death associated with accreditation status in different models

| **Model 1: Unadjusted^a^** | | | |  | **Model 2: Fully adjusted^b^** | | |  | **Model 3: IPTW models^c^** | | |
| --- | --- | --- | --- | --- | --- | --- | --- | --- | --- | --- | --- |
| **Pre-accreditation** | **Post-accreditation** | | |  | **Post-accreditation** | | |  | **Post-accreditation** | | |
|  | **Year 1** | **Year 2** | **Year 2+** |  | **Year 1** | **Year 2** | **Year 2+** |  | **Year 1** | **Year 2** | **Year 2+** |
| 1st readmission for worsening HF or CV death | | | | | | | | | | | |
| Reference | .850 (.830–.869) | .810 (.789–.830) | .674 (.656–.693) |  | .893 (.871–.916) | .855 (.830–.880) | .720 (.695–.745) |  | .829 (.810–.849) | .812 (.791–.834) | .713 (.691–.736) |
|  | < .0001 | < .0001 | < .0001 |  | < .0001 | < .0001 | < .0001 |  | < .0001 | < .0001 | < .0001 |
| 1st readmission for worsening HF | | | | | | | | | | | |
| Reference | .835 (.812–.857) | .814 (.790–.839) | .623 (.603–.644) |  | .865 (.841–.891) | .879 (.850–.910) | .719 (.690–.749) |  | .798 (.777–.821) | .834 (.809–.860) | .691 (.666–.718) |
|  | < .0001 | < .0001 | < .0001 |  | < .0001 | < .0001 | < .0001 |  | < .0001 | < .0001 | < .0001 |
| CV death | | | | | | | | | | | |
| Reference | .899 (.870–.928) | .853 (.824–.884) | .747 (.720–.777) |  | 1.008 (.972–1.045) | .942 (.904–.983) | .820 (.781–.862) |  | .926 (.895–.957) | .879 (.846–.913) | .768 (.734–.803) |
|  | < .0001 | < .0001 | < .0001 |  | .6688 | .0055 | < .0001 |  | < .0001 | < .0001 | < .0001 |
| ^a^Univariate logistic regression models. ^b^Adjusted for age, sex, sites, admission year, LVEF, comorbid conditions (prior MI, diabetes, hypertension, AF, renal insufficiency, COPD, anemia), ACEi/ARB/ARNI, beta-blockers, MRA. ^c^IPTW propensity score outcome models  ACEi, angiotensin-converting enzyme inhibitor; AF, atrial fibrillation; ARB, angiotensin receptor blocker; ARNI, angiotensin receptor–neprilysin inhibitor; CI, confidence interval; COPD, chronic obstructive pulmonary disease; CV, cardiovascular; HF, heart failure; IPTW, inverse probability of treatment weight; LVEF, left ventricular ejection fraction; MI, myocardial infarction; MRA, mineralocorticoid receptor antagonist; OR, odds ratio | | | | | | | | | | | |

## Supplementary Table S13 Sensitivity analysis - Odd ratios (95% CI) of the 1-year composite endpoint of readmissions for worsening HF or CV death associated with accreditation status, time-adjusted for PCI, CABG, and hospital level (tertiary/secondary)

|  |  |  |  | **Model 1** | |  | | **Model 2** | | |  |
| --- | --- | --- | --- | --- | --- | --- | --- | --- | --- | --- | --- |
| **Primary outcome** | **No. of events** | **No. of patients** | **Rate (%)** | **OR (95% CI)** | ***P*-values** | |  | | **OR (95% CI)** | ***P*-values** | |
| Pre-accreditation | 19 380 | 128 740 | 15.1% | **Reference** |  | |  | | **Reference** |  | |
| Year 1 | 1 5309 | 116 975 | 13.1% | 0.893 (0.871–0.916) | <.0001 | |  | | 0.900 (0.88–0.921) | <.0001 | |
| Year 2 | 10 632 | 84 734 | 12.5% | 0.855 (0.83–0.88) | <.0001 | |  | | 0.869 (0.847–0.892) | <.0001 | |
| Year 2+ | 8286 | 77 624 | 10.7% | 0.72 (0.695–0.745) | <.0001 | |  | | 0.750 (0.729–0.772) | <.0001 | |
| Model 1: Adjusted for age, gender, region, admission year, LVEF, ln(NT-proBNP), comorbid conditions (prior MI, diabetes, hypertension, AF, renal insufficiency, COPD, anaemia), RASi, beta-blocker, MRA, as appropriate. Model 2: covariates in model1 + PCI, CABG, and hospital (tertiary/secondary hospitals)  AF, atrial fibrillation; CABG, coronary artery bypass grafting; CI, confidence interval; COPD, chronic obstructive pulmonary disease; CV, cardiovascular; HF, heart failure; LVEF, left ventricular ejection fraction; MI, myocardial infarction; MRA, mineralocorticoid receptor antagonist; NT-proBNP, *N*-terminal pro-B-type natriuretic peptide; OR, odds ratio; PCI, percutaneous coronary intervention | | | | | | | | | | |  |

## Supplementary Table S14 Sensitivity analysis - OR (95% CI) of the 1-year composite endpoint of readmissions for worsening HF or CV death associated with accreditation status, where patients without echocardiography were excluded

| **Primary outcome** | **No. of events** | **No. of patients** | **Rate (%)** | **OR (95% CI)** | ***P*-values** |
| --- | --- | --- | --- | --- | --- |
| Pre-accreditation | 15 814 | 108 223 | 14.61% | **Reference** |  |
| Year 1 | 13 648 | 106 294 | 12.84% | 0.898 (0.876–0.921) | <.0001 |
| Year 2 | 9414 | 76 406 | 12.32% | 0.858 (0.834–0.882) | <.0001 |
| Year 2+ | 7466 | 71 477 | 10.45% | 0.714 (0.693–0.735) | <.0001 |
| CI, confidence interval; CV, cardiovascular; HF, heart failure; OR, odds ratio | | | | | |

## Supplementary Table S15 Comparison of health status among telephone follow-ups and clinic visits

| **Follow-up model** | T+C+ | T-C+ | T+C- | T-C- |
| --- | --- | --- | --- | --- |
| **n** | 20 129 | 39 311 | 178 434 | 170 199 |
| **Demographic** |  |  |  |  |
| Female, % | 39.1 | 39.4 | 41.6 | 41.2 |
| Age (y), mean ± SD | 68.6±13.3 | 68.7±13.4 | 69.5±13.2 | 69.8±13.4 |
| **History, comorbidities, %** |  |  |  |  |
| Hypertension | 58.4 | 59.8 | 58.3 | 57.6 |
| Type 2 diabetes mellitus | 28.2 | 28.4 | 26.8 | 27.5 |
| Hyperlipidaemia | 14.1 | 15.2 | 13.4 | 15.4 |
| Obesity | 20.1 | 17.3 | 19.0 | 18.0 |
| Ischemic heart disease | 63.1 | 62.2 | 62.3 | 60.1 |
| Prior myocardial infarction | 24.2 | 24.1 | 22.3 | 23.3 |
| Atrial fibrillation | 34.4 | 34.2 | 32.2 | 33.5 |
| Stroke | 11.3 | 11.5 | 11.7 | 13.1 |
| Renal insufficiency | 13.0 | 13.3 | 12.5 | 12.3 |
| COPD or asthma | 9.0 | 8.3 | 9.5 | 10.0 |
| Anemia | 19.9 | 20.5 | 19.8 | 23.0 |
| **History, device/procedure, %** |  |  |  |  |
| Prior PCI | 18.1 | 22.7 | 22.1 | 19.2 |
| Prior CABG | 0.8 | 0.6 | 0.6 | 1.0 |
| Prior Pacemaker | 4.0 | 3.7 | 3.0 | 3.8 |
| Prior ICD | 1.2 | 1.3 | 0.6 | 0.9 |
| Prior CRT-P | 0.3 | 0.3 | 0.2 | 0.4 |
| Prior CRT-D | 1.1 | 1.1 | 0.6 | 0.8 |
| **Vitals (closest to admission)** |  |  |  |  |
| BMI (kg/m^2^) | 23.7±3.7 | 23.7±3.6 | 23.6±3.7 | 23.6±3.6 |
| SBP (mmHg) | 131.6±23.9 | 131.7±24.2 | 131.1±24.4 | 131.1±24.2 |
| DBP (mmHg) | 77.9±14.1 | 77.6±13.9 | 78±14.2 | 77.3±14 |
| Heart rate (bpm) | 81.6±20.6 | 81.6±19.7 | 82.5±20.2 | 83.6±20.7 |
| **Laboratory (closest to admission)** |  |  |  |  |
| NT-proBNP (pg/mL) | 1885.1  (789.0–4378.5) | 1869.0  (787.6–4371.0) | 2176.0  (878.6–5366.0) | 2479.4  (980.0–6100.0) |
| LVEF (%) | 46.6±13.7 | 46.7±13.9 | 46.6±13.5 | 46.7±13.9 |
| T+C+: Telephone follow-up>2 times/year and Clinic visits>2 times/year  T-C+: Telephonic follow-up<=2 times/year and Clinic visits>2 times/year  T+C-: Telephonic follow-up>2 times/year and Clinic visits<=2 times/year  T-C-: Telephonic follow-up<=2 times/year and Clinic visits<=2 times/year  BMI, body mass index; bpm; beats per minute; CABG, coronary artery bypass grafting; COPD, chronic obstructive pulmonary disease; CRT-D, cardiac resynchronization therapy defibrillator; CRT-P, cardiac resynchronization therapy pacemaker; DBP, diastolic blood pressure; ICD, implantable cardioverter defibrillator; LVEF, left ventricular ejection fraction; NT-proBNP, *N*-terminal pro-B-type natriuretic peptide; PCI, percutaneous coronary intervention; SBP, systolic blood pressure; SD, standard deviation. | | | | |
